# Analysis of High-Dose Ascorbate-Induced Cytotoxicity in Human Glioblastoma Cells and the Role of Dehydroascorbic Acid and Iron

**DOI:** 10.3390/antiox13091095

**Published:** 2024-09-10

**Authors:** Alban Piotrowsky, Markus Burkard, Katharina Hammerschmidt, Hannah K. Ruple, Pia Nonnenmacher, Monika Schumacher, Christian Leischner, Susanne Berchtold, Luigi Marongiu, Thomas A. Kufer, Ulrich M. Lauer, Olga Renner, Sascha Venturelli

**Affiliations:** 1 Department of Nutritional Biochemistry, Institute of Nutritional Sciences, University of Hohenheim, Garbenstrasse 30, 70599 Stuttgart, Germany; 2 Department of Medical Oncology and Pneumology, Virotherapy Center Tuebingen (VCT), Medical University Hospital, 72076 Tuebingen, Germany; 3 HoLMiR-Hohenheim Center for Livestock Microbiome Research, University of Hohenheim, Garbenstrasse 30, 70599 Stuttgart, Germany; 4 Department of Immunology, Institute of Nutritional Medicine, University of Hohenheim, Fruwirthstrasse 12, 70593 Stuttgart, Germany; 5 German Cancer Consortium (DKTK), Partner Site Tuebingen, a Partnership between DKFZ and University Hospital Tuebingen, 72076 Tuebingen, Germany; 6 Faculty of Food and Nutrition Sciences, Hochschule Niederrhein, University of Applied Sciences, Rheydter Strasse 277, 41065 Moenchengladbach, Germany; 7 Department of Vegetative and Clinical Physiology, Institute of Physiology, University of Tuebingen, Wilhelmstrasse 56, 72074 Tuebingen, Germany

**Keywords:** ascorbate, vitamin C, glioblastoma, cancer, iron, reactive oxygen species

## Abstract

Several studies have demonstrated, both in vitro and in animal models, the anti-tumor efficacy of high-dose ascorbate treatment against a variety of tumor entities, including glioblastoma, the most common and aggressive primary malignant brain tumor. The aim of this study was to investigate the effects of high-dose ascorbate as well as dehydroascorbic acid on human glioblastoma cell lines and to evaluate different treatment conditions for the combined administration of ascorbate with magnesium (Mg^2+^) and iron (Fe^3+^). Intracellular levels of reactive oxygen species and the induction of cell death following ascorbate treatment were also investigated. We demonstrated high cytotoxicity and antiproliferative efficacy of high-dose ascorbate in human glioblastoma cells, whereas much weaker effects were observed for dehydroascorbic acid. Ascorbate-induced cell death was independent of apoptosis. Both the reduction in cell viability and the ascorbate-induced generation of intracellular reactive oxygen species could be significantly increased by incubating the cells with Fe^3+^ before ascorbate treatment. This work demonstrates, for the first time, an increase in ascorbate-induced intracellular ROS formation and cytotoxicity in human glioblastoma cells by pre-treatment of the tumor cells with ferric iron, as well as caspase-3 independence of cell death induced by high-dose ascorbate. Instead, the cell death mechanism caused by high-dose ascorbate in glioblastoma cells shows evidence of ferroptosis. The results of the present work provide insights into the efficacy and mode of action of pharmacological ascorbate for the therapy of glioblastoma, as well as indications for possible approaches to increase the effectiveness of ascorbate treatment.

## 1. Introduction

High-dose ascorbate (vitamin C) has different anti-tumor functions. Studies investigating ascorbate alone or in combination with other anticancer agents describe potential mechanisms of action such as epigenome regulation, oxygen-sensing, immunomodulatory functions, inhibition of epithelial-to-mesenchymal transition, and regulation of kinase activity [1]. However, its pro-oxidant activity is most commonly described. In tumor cells, the pro-oxidant mechanism of ascorbate is based on its redox chemistry with metals, which allows increased formation of oxygen radicals [2]. The specific sensitivity of cancer cells to high concentrations of ascorbate is characterized by reduced activity of catalytic enzymes degrading these reactive oxygen species (ROS) and therefore regulating radical levels [3]. A special role is also played by iron. In the case of increased iron demand and iron deficiency, external iron supply increases the amount of intracellular free and unbound iron (ferrous iron, Fe^2+^), also called labile iron pool (LIP), thus potentiating ROS formation [4,5,6]. Magnesium, on the other hand, appears to influence the activity of the sodium-dependent vitamin C transporter 2 (SVCT2) and thus regulates ascorbate uptake into the cell [7].

The ability of high-dose ascorbate to suppress tumor cell growth has also been described for glioblastoma in vitro [4], in animals [6], and in a clinical trial [4,8] in which patients were treated with standard therapy and additional high-dose ascorbate administration. Gliomas are composed of heterogenous cell lineages, such as astrocytes, oligodendrocytes, and ependymal cells; glioblastomas, as the most malignant astrocytoma, are the most common primary malignant brain tumors [9]. Displaying a highly invasive phenotype and the ability to develop resistance to the chemotherapeutic temozolomide (TMZ), glioblastoma belongs to the six most aggressive tumors [10]. Under standard treatment, established by Stupp et al., including a combination of surgical resection and combined radio- and TMZ chemotherapy, the prognosis for patients with this disease is still poor, with a median overall survival (OS) of 14–16 months [11,12]. Besides recent extensive efforts to better understand the pathomechanisms and the development of some promising novel therapeutic approaches in the fields of chemotherapy, molecular targeted therapy, and immunotherapy, none of these treatment innovations has yet been able to surpass the efficacy of Stupp’s protocol, the standard of glioblastoma therapy since 2005 [12]. In addition, unlike other solid tumors, the physiological conditions such as the blood-brain-barrier (BBB) and the unique tumor and immune microenvironment pose significant challenges to the development of novel therapies [13], and pre-existing or acquired drug resistance is one of the biggest obstacles in glioblastoma treatment [14]. Due to the only incremental therapeutic advances, glioblastoma remains incurable, with a dismal 5-year survival rate of still less than 10% [15], which confirms the urgent need for better therapeutic strategies.

The demonstrated benefit of combining high-dose ascorbate treatment with standard therapy is therefore a promising starting point for further detailed research. For instance, in non-small cell lung cancer (NSCLC), there is strong evidence for a predominant role of ascorbate rather than its oxidized form dehydroascorbic acid (DHA) [4]. Most glioblastomas originate from astrocytes that can only use DHA via a bystander effect but not ascorbate under physiological conditions [16]. Therefore, the question remains open as to which is the main active substance and the corresponding mechanism behind the high-dose ascorbate treatment in glioblastomas and whether the efficacy is mainly mediated by pro-oxidative effects.

The aim of the present work was (i.) to compare the effects of high-dose ascorbate and DHA on different glioblastoma cell lines, and (ii.) to show whether iron (Fe^3+^) and magnesium (Mg^2+^) cations can enhance ascorbate-induced toxicity and suppress glioblastoma cell viability. In addition, (iii.) suitable treatment conditions for the combined administration of ascorbate and iron were evaluated, and (iv.) apoptosis as a possible ascorbate-induced cell death mechanism was investigated.

## 2. Materials and Methods

### 2.1. Cell Culture and Reagents

Human-derived glioblastoma cell lines SF268, SF295, and U251 were selected from the US National Cancer Institute’s NCI-60 tumor cell panel, which was purchased from Charles River Laboratories (Charles River Laboratories Inc., New York, NY, USA). NHEK-Ad cells were obtained from Lonza Group AG (Catalog No. 00192627, Basel, Switzerland) and CCD-18Co cells from ATCC (CRL-1459, Manassas, VA, USA). Glioblastoma cells were cultured in RPMI-1640 medium (Thermo Fisher Scientific Inc., Waltham, MA, USA) containing 10% fetal calf serum (FCS, Merck KGaA, Darmstadt, Germany) and 1% penicillin/streptomycin (Thermo Fisher Scientific Inc.). NHEK-Ad cells were cultured in EpiLife medium (MEPI500CA, Invitrogen, Waltham, MA, USA) under addition of the HKGS Kit (S001K, Invitrogen) and CCD-18Co cells in MEM (Thermo Fisher Scientific Inc.) with 10% FCS (Merck KGaA). All cell lines were cultivated at 37 °C in a humidified atmosphere containing 5% CO_2_. All experiments were performed with cells from passages 11–25.

Ascorbate (Pascorbin^®^) was purchased from Pascoe pharmazeutische Praeparate GmbH (CAS 50-81-7, Giessen, Germany). Staurosporine (STS) and RAS-selective lethal 3 (RSL3) were obtained from MedChemExpress (STS: CAS 62996-74-1; RSL: 1219810-16-8, Monmouth Junction, NJ, USA), and DHA from Acros Organics (96% purity, CAS 490-83-5, Geel, Belgium). Chloroquine (CQ) and rapamycin (RAPA) were obtained from Enzo Biochem, Inc. (ENZ-51031, Farmingdale, NY, USA). Triton^®^ X-100, tert-butyl hydroperoxide (TBH), sulforhodamine B (SRB), and 4-methylumbeliferyl heptanoate (MUH) were purchased from Sigma-Aldrich (Triton^®^ X-100: CAS 9002-93-1; TBH: CAS 75-91-2, SRB: CAS 3520-42-1, MUH: CAS 18319-92-1, St. Louis, MO, USA). Deferoxamine mesylate (DFO) was obtained from Selleck Chemicals LLC (Catalog No. S5742, Houston, TX, USA).

### 2.2. Cell Viability Assays

To investigate the cytotoxicity of high-dose ascorbate and DHA against human glioblastoma cells, MUH and SRB assays were performed as previously described [17]. Cells were seeded at a density of 4 × 10^4^ (SF268, NHEK-Ad, and CCD-18Co), 3 × 10^4^ (SF295), or 2 × 10^4^ (U251) cells per well in a 24-well format. To investigate the effect of pre-incubation with Fe^3+^ or Mg^2+^, 100 µM FeCl_3_ or 5 mM MgCl_2_ were added to the medium at the time of seeding. Then, 24 h after seeding, cells were washed twice with phosphate-buffered saline (PBS: 8.09 mM Na_2_HPO_4_, 2.68 mM KCl, 1.47 mM KH_2_PO_4_, 0.14 M NaCl, pH 7.4) and treated with different concentrations of ascorbate (0.2 mM, 0.4 mM, 0.6 mM, 0.8 mM, 1.0 mM, 2.0 mM, and 4.0 mM) or DHA (0.2 mM, 1.0 mM, 2.0 mM, and 4.0 mM) in duplicates. To investigate co-incubation with iron or magnesium, 100 µM FeCl_3_ or 5 mM MgCl_2_ were added to the medium at the same time as ascorbate. As a positive control, cells were treated with 0.1% Triton^®^ X-100 for 15 min. To investigate the effect of DFO on ascorbate treatment, the cells were treated simultaneously with 50 µM DFO and the corresponding ascorbate concentrations for 24 h.

To perform the MUH assay, cells were washed with PBS after 24 h of ascorbate treatment and 400 µL of MUH solution (100 µg/mL in phenol red-free, FCS-free RPMI-1640 medium) was pipetted into each well. After incubation at 37 °C for 1 h, the fluorescence intensity was detected at 460 nm with the Synergy^TM^ H1 microplate reader (BioTek Instruments, Inc., Winooski, VT, USA). The intensity of fluorescence is an indicator of the number of viable cells, and this was used to calculate the percentage of cells remaining after treatment compared with the respective controls.

Subsequently, the SRB assay was performed. After treatment, cells were placed on ice and washed twice with cold PBS (4 °C) immediately after the measurement of the MUH assay. The cells were then fixed with 10% trichloroacetic acid (TCA, Carl Roth GmbH & Co. KG, Karlsruhe, Germany) for 30 min at 4 °C. After fixation, TCA was discarded, and the cells were washed four times with deionized water. The plates were dried overnight at 40 °C in a drying oven. The next day, the cells were stained with 200 µL of SRB staining solution for 10 min at room temperature, the staining solution was discarded, and the cells were washed with deionized water until the effluent was colorless. The plates were dried again overnight at 40 °C. The remaining dye was then dissolved by adding 500 µL of Tris base (1 mM, pH 10.5) and incubated for 10 min at room temperature on a platform shaker. The contents of each well were transferred in triplicates of 80 μL each to a 96-well microassay plate, and the optical density was measured in a microplate reader at 520 nm and 620 nm. Data represent the mean of the optical density values relative to untreated control cells. Three independent experiments were carried out.

### 2.3. Investigation of Cell Proliferation

To investigate the antiproliferative effect of high-dose ascorbate, cell number determination was performed using the Lionheart FX automated microscope (BioTek Instruments, Inc.). Cells were seeded in 24-well format at a cell density of 4 × 10^4^ (SF268), 3 × 10^4^ (SF295), or 2 × 10^4^ (U251) cells per well and incubated in the Lionheart FX at 37 °C and 5% CO_2_. Images of four defined sections of each well were taken hourly over a 72-h period. After 24 h, cells were treated with ascorbate concentrations up to 4 mM in duplicates and incubated for an additional 48 h and photographed hourly. Triton^®^ X-100 (0.1%) was used as a positive control. Cell number was subsequently determined with the captured images by using Gen5 software version 3.10 (BioTek Instruments, Inc.). At all time points, the cell number on the four acquired images of each well was determined, summed (total area: 0.108407 cm^2^), and the cell number per well (area: 1.9 cm^2^) was calculated by multiplying by a factor of 17.5265.

### 2.4. Investigation of Intracellular ROS Levels

Cells were seeded at a density of 4 × 10^4^ (SF268), 3 × 10^4^ (SF295), or 2 × 10^4^ cells (U251) in 24-well format. To investigate the effect of pre-incubation with iron, 100 µM FeCl_3_ was added to the medium at seeding. After 24 h, cells were washed twice with PBS and treated with ascorbate concentrations up to 2 mM. After 3 h, 4 h, 5 h, or 6 h of treatment, dichlorodihydrofluorescein diacetate (DCFH-DA) assay was performed to measure intracellular ROS induction. As a positive control, cells were treated with TBH (1 mM) for 3 h. Cells were harvested at the indicated time points, duplicates were combined, the cell pellet was washed twice with PBS, and then incubated for 30 min in 5 µM DCFH-DA in phenol red-free, FCS-free RPMI-1640 medium at 37 °C in the dark. The supernatant was discarded, and the cells were washed again with PBS and resuspended in 400 µL of phenol red-free, FCS-free RPMI-1640 medium. This was followed by analysis of the samples with absorbance at 488 nm and detection of emission at 530 nm using a NovoCyte^®^ 2060R flow cytometer (Agilent Technologies, Santa Clara, CA, USA) using NovoExpress^®^ software version 1.4.1 (ACEA, Biosciences, Inc., San Diego, CA, USA). For this purpose, 10,000 events per sample were measured. Three independent experiments were performed.

### 2.5. Analysis of Cell Cycle with Propidium Iodide Staining by Flow Cytometry

For cell cycle analysis, cells were seeded at densities of 8 × 10^4^ (SF268), 6 × 10^4^ (SF295), and 4 × 10^4^ (U251) on a 12-well plate and incubated for 24 h. Subsequently, the cells were treated with ascorbate concentrations up to 1 mM for 24 h. Treatment with STS at a concentration of 5 µM for 20 h was used as a positive control. After treatment, cells were harvested, washed with PBS, and fixed with 70% ice-cold ethanol overnight at 4 °C. Cells were again washed twice with PBS and incubated in PBS with propidium iodide (PI, 50 µg/mL, Sigma-Aldrich) and RNAse (100 µg/mL, Roche Diagnostics, Mannheim, Germany) for at least 20 min at 4 °C. Subsequently, the distribution of cells in the different cell cycle phases was detected by flow cytometry (NovoCyte^®^ 2060R flow cytometer, Agilent Technologies). In total, 10,000 events were recorded per measurement and three independent experiments were performed.

### 2.6. Morphological Cell Nucleus Examination

Morphological changes in cell nuclei were examined after ascorbate treatment. Cells were seeded at a density of 1.72 × 10^4^ (SF268), 1.29 × 10^4^ (SF295), or 0.86 × 10^4^ (U251) in the chambers of 8-well cell imaging slides. After 24 h, cells were washed twice with PBS and treated with 1 mM ascorbate or 10 µM STS as a positive control for 6 h in phenol red-free, FCS-free RPMI-1640 medium. Live and dead cells were stained by adding the Live Green and Dead Red dyes (LIVE/DEAD^TM^ Cell Imaging Kit, Thermo Fisher Scientific Inc.) 2 h after the start of treatment according to the manufacturer’s protocol. After treatment, nuclei were stained with 1 µg/mL Hoechst 33342 (Sigma-Aldrich) for 20 min at 37 °C. Fluorescence images were then acquired using a Lionheart FX microscope (BioTek Instruments) with the 20× objective lens Two independent experiments were performed.

### 2.7. Immunoblotting

SF268 cells (2 × 10^5^ cells/well) were seeded in 6-well plates and treated with different ascorbate concentrations (0.2 mM, 0.4 mM, 0.6 mM, 0.8 mM, and 1.0 mM) on the following day. For this process, 5 µM and 10 µM STS served as positive control for apoptosis, 1 µM RSL3 served as positive control for ferroptosis, and the combination of 60 µM CQ with 500 nM RAPA served as a positive control for autophagy. After 6 h of treatment (for analysis of cleaved caspase 3) or 8 h of treatment (for glutathion peroxidase 4 (GPX4), transferrin receptor 1 (TfR1) and microtubule-associated proteins 1A/1B light chain 3B (LC3B) analysis), the cells were washed twice with PBS, harvested, and resuspended in 50 µL lysis buffer (150 mM NaCl, 50 mM Tris-HCl pH 7.4, 1% Triton^®^ X-100, 0.5% sodium deoxycholate, 0.1% sodium dodecyl sulfate (SDS), 10 µL/mL aprotinin, 10 µL/mL leupeptin, 10 µL/mL pepstatin A, 1.5 µL/mL phenylmethanesulfonyl fluoride (PMSF), 1 µL/mL Na_3_VO_4_). After incubation of the cells in lysis buffer on ice for 30 min, the lysates were purified by centrifugation at 13,000 rpm at 4 °C for 20 min. The supernatant was collected, and the lysates were stored at −20 °C. The protein concentration of the lysates was determined in triplicates using the protein determination method according to Lowry. For each lysate, 35 µg protein was mixed with Laemmli buffer (250 nM Tris-HCl pH 6.8, 40% glycerol, 8% SDS, 20% 2-mercaptoethanol, crumb bromophenol blue), boiled at 95 °C for 5 min, and the proteins were separated in 10% SDS-polyacrylamide gels by electrophoresis. Proteins were then blotted onto a polyvinylidene difluoride (PVDF) membrane, blocked in 5% milk powder in Tris-buffered saline containing 0.1% Tween-20 (TBST) for 1 h at room temperature and incubated with the primary antibody (anti-Cleaved Caspase-3: ab2302, 1:1000 in 5% bovine serum albumin (BSA), Abcam, Cambridge, UK, anti-GPX4: #52455, 1:1000 in 5% BSA, anti-TfR1: #13113, 1:1000 in 5% BSA, or anti-LC3B: #2775, 1:1000 in 5% BSA, Cell Signaling Technology, Danvers, MA, USA) overnight at 4 °C. After washing three times with TBST, the membranes were incubated for 45 min at room temperature with the secondary antibody (goat anti-rabbit IgG, horseradish peroxidase (HRP)-coupled: 7074s, 1:10,000 in 5% milk powder in TBST; Cell Signaling Technology Inc., Danvers, MA, USA) and washed again three times with TBST. Subsequently, the proteins were detected by the WesternBright chemiluminescence substrate Sirius (Biozym Scientific GmbH, Hessisch Oldendorf, Germany) in the Fusion FX chemiluminescence detector (Vilber, Collégien, France).

### 2.8. Statistical Analysis

Statistical analysis of data was performed using GraphPad Prism version 9.0 (GraphPad Software, San Diego, CA, USA). For multiple group comparisons, one-way ANOVA with subsequent Dunnett’s multiple comparisons test was used for *p*-value calculation and significance determination. *p*-values ≤ 0.05 were considered statistically significant (*: *p* ≤ 0.05; **: *p* ≤ 0.01; ***: *p* ≤ 0.001).

## 3. Results

### 3.1. Ascorbate but Not DHA Induces Cytotoxic Effects in Glioblastoma Cells

**Figure 1:**
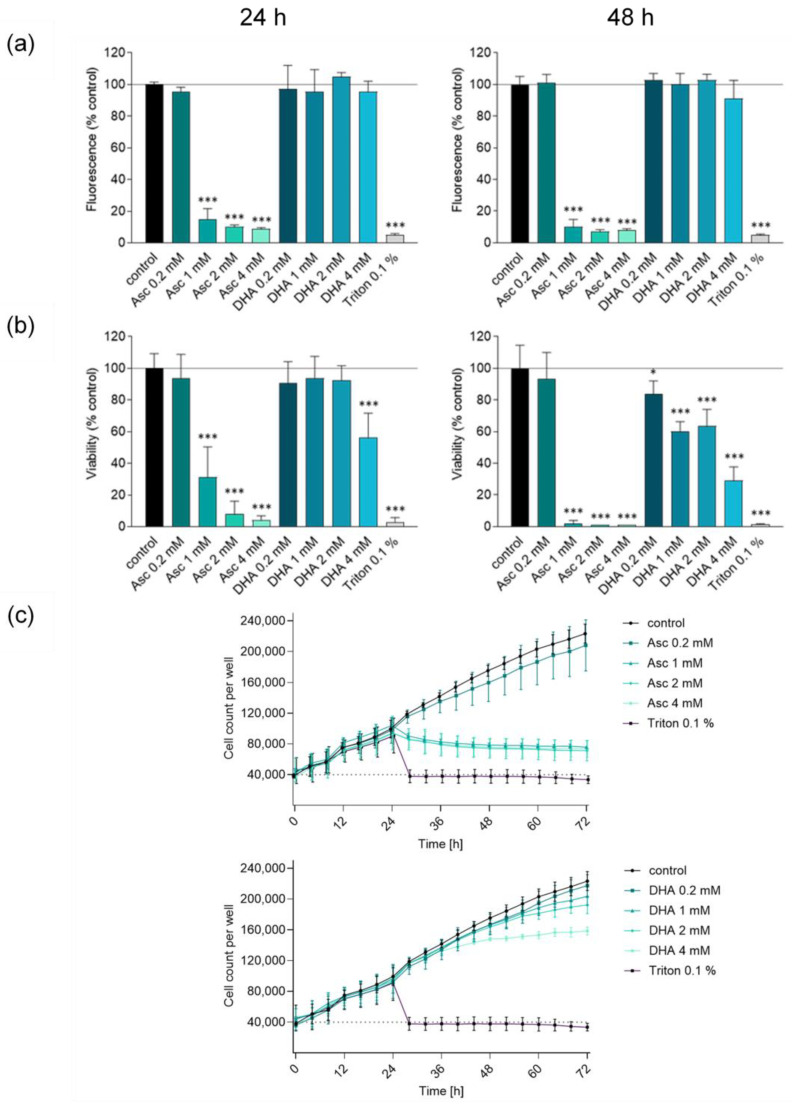
Evaluation of the sensitivity of human glioblastoma cells to high-dose ascorbate and DHA treatment. SF268 cells were treated for 24 h or 48 h with different concentrations of Asc or DHA (0.2 mM, 1 mM, 2 mM, and 4 mM). Triton X-100 at 0.1% (*v*/*v*) served as a positive control. Cell viability was assessed by MUH assay (**a**) and SRB assay (**b**). Results are presented as percentage of fluorescence intensity and viability, respectively, compared to the untreated control. (**c**) Antiproliferative effects were detected by cell number determination using the Lionheart FX automated microscope (BioTek Instruments, Inc., Winooski, VT, USA). Treatment was performed 24 h after seeding. Results are presented as cell number per well every 4 h. Three independent experiments were performed, each in duplicates. Error bars represent mean ± SD, statistical analysis with one-way ANOVA and subsequent Dunnett’s multiple comparisons test, confidence interval 95%. *: *p* ≤ 0.05; ***: *p* ≤ 0.001. Asc, ascorbate; DHA, dehydroascorbic acid; MUH, 4-methylumbelliferyl heptanoate; SRB, sulforhodamine B.

Schoenfeld et al. reported that glioblastoma can be effectively treated with high-dose vitamin C [4]. In NSCLC, there is clear evidence for the predominant effect of ascorbate. Since most glioblastomas originate from astrocytes, they take up DHA but not ascorbate and regenerate it to form ascorbate; as such, the question arises as to which substance (ascorbate versus DHA) plays the crucial cytotoxic role [18]. The cytotoxic effects of the two forms of vitamin C were tested at two different incubation times (24 h and 48 h) and at four different concentrations (0.2 mM, 1 mM, 2 mM, and 4 mM). The sensitivity of glioblastoma cells to high dose vitamin C (Asc) was thereby verified by three different assays (MUH, SRB, and by cell count). Representative data are shown for SF268 cells (Figure 1a–c). Evaluation of the measurements for SF295 and U251 glioblastoma cell lines confirmed the trends and statements presented for SF268 without contradiction (Supplementary Figures S1 and S2). As little as 1 mM ascorbate was able to significantly suppress the viability of glioblastoma cells to below 20% or less after 24 h of treatment compared to the untreated control (Figure 1a). In contrast, the decrease in viability for DHA was only observed up to 50% from 4 mM after 24 h incubation with the SRB assay and better detectable after 48 h of incubation, while almost no effect was detected in the MUH assay (Figure 1a,b). However, the DHA-mediated effect was evident, though less pronounced than the cytotoxicity induced by ascorbate. These results were confirmed by continuous cell count analyses over a time period of 72 h (Figure 1c). Based on these experiments, the suppression of viability associated with ascorbate was further investigated, and the 48 h incubation period was no longer followed, as the full effect of ascorbate cytotoxicity was already detectable after 24 h.

In contrast, treatment of the human colon cell line with fibroblast morphology CCD-18Co caused no decrease in cell viability measured by MUH and SRB assays at ascorbate concentrations up to and including 2 mM. Treatment of the human epidermal keratinocyte cell line NHEK-Ad resulted in only a slight decrease in cell viability after treatment with high pharmacological ascorbate concentrations, which indicates the safety of therapeutic intravenous high-dose ascorbate treatment (Supplementary Figure S3).

### 3.2. Effect of Iron and Magnesium on Ascorbate-Induced Cytotoxicity

To confirm the iron-dependence of malignant cells and to demonstrate the effect of increasing levels of free iron, the combination of high-dose ascorbate and iron was investigated. However, there is also evidence from previous studies that there is a difference between co-incubation and pre-incubation with iron [19,20,21,22,23]. Furthermore, a recent report by Cho et al. described enhanced anticancer effects of ascorbate in colorectal and breast cancer cells by the addition of magnesium, which is known to enhance transporter-mediated ascorbate uptake [24].

**Figure 2:**
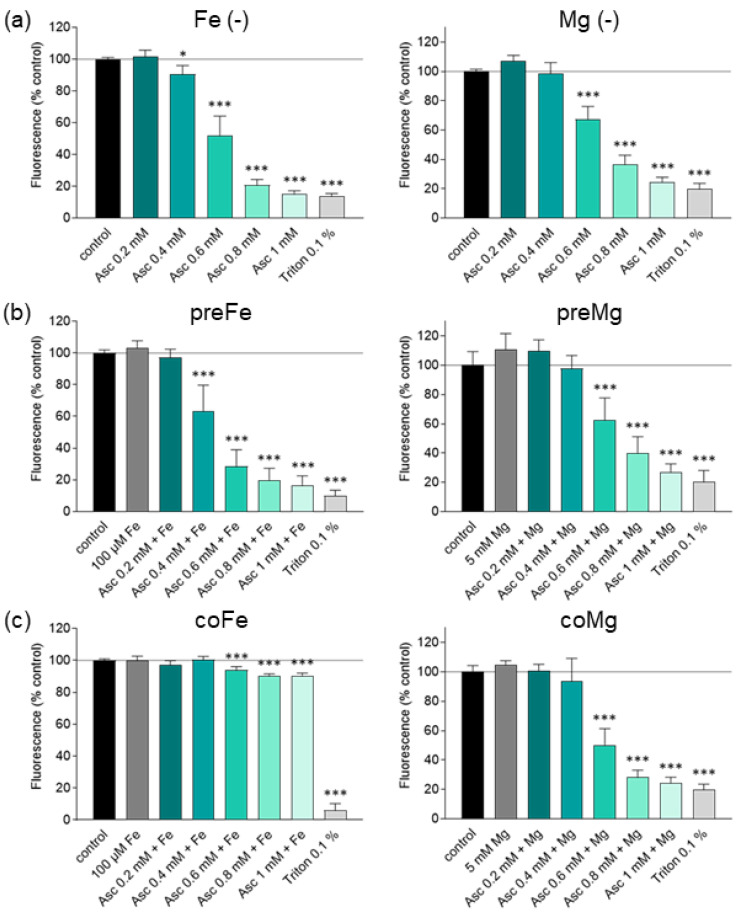
Effect of iron and magnesium on ascorbate-induced cytotoxicity in human glioblastoma cells shown by MUH assay. SF268 cells were treated with different concentrations of Asc for 24 h (0.2 mM, 0.4 mM, 0.6 mM, 0.8 mM, and 1 mM). Triton X-100 at 0.1% (*v*/*v*) served as a positive control. Cells were either treated with Asc alone (**a**), pre-incubated with FeCl_3_ (100 µM) or MgCl_2_ (5 mM) for 24 h immediately prior to Asc treatment (**b**), or co-incubated with Asc and FeCl_3_ or MgCl_2_ (**c**). Results are presented as percentage of fluorescence intensity compared to the untreated control. Three independent experiments were performed, each in duplicates. Error bars represent mean ± SD, statistical analysis with one-way ANOVA and subsequent Dunnett’s multiple comparisons test, confidence interval 95%. *: *p* ≤ 0.05; ***: *p* ≤ 0.001. Asc, ascorbate; co, co-incubation; Fe, ferric chloride (FeCl_3_); Mg, magnesium chloride (MgCl_2_); MUH, 4-methylumbelliferyl heptanoate; pre, pre-incubation.

To test the potentiating role of both cations, cells were pre- or co-incubated for 24 h with 100 µM FeCl_3_ or 5 mM MgCl_2_, in addition to ascorbate treatment. Building on the results collected in the first results section (Figure 1a–c), the concentration range from 0.2 mM to 1 mM ascorbate was investigated more in detail. An iron concentration of 100 µM FeCl_3_ was chosen according to a physiologically achievable in vivo value [20,23]. In preliminary studies, glioblastoma cells were treated with different concentrations of magnesium (1–10 mM) to rule out any cytotoxic reactions. Consistent with data in colorectal and breast cancer [24], glioblastoma cells showed no cytotoxicity at any of the concentrations tested. Therefore, 5 mM MgCl_2_ was used as a reference value. Overall, exposure of glioblastoma cells (SF268, SF295, and U251) to increasing doses of ascorbate in combination with cations showed a dose-dependent reduction in cell viability measured by MUH assay (Figure 2a–c, Supplementary Figures S4 and S5). In two of the three cell lines (SF268 and U251), ascorbate-induced toxicity was markedly enhanced by pre-incubation with iron compared with ascorbate treatment alone. Starting with 0.4 mM ascorbate alone, cell viability of SF268 was reduced by 9%, whereas with 0.4 mM ascorbate and pre-incubation with 100 µM FeCl_3_, a reduction to 63% compared to the untreated control was achieved (Figure 2a–c). The benefit of pre-incubation was also evident at the other concentrations tested and was confirmed by the SRB assay (Supplementary Figures S6–S8). In line with data shown for breast, colon, and lung cancer [22,23], we have demonstrated that co-treatment with iron impairs the ascorbate-induced toxicity in glioblastoma cells. Irrespective of the treatment regimen, incubation with ascorbate and magnesium showed no increased efficacy compared to ascorbate alone in glioblastoma cells (Figure 2a–c). These findings were confirmed by MUH and SRB assays (Supplementary Figure S6), respectively.

### 3.3. Potentiation of ROS Formation by Ascorbate and Iron Pre-Treatment

**Figure 3:**
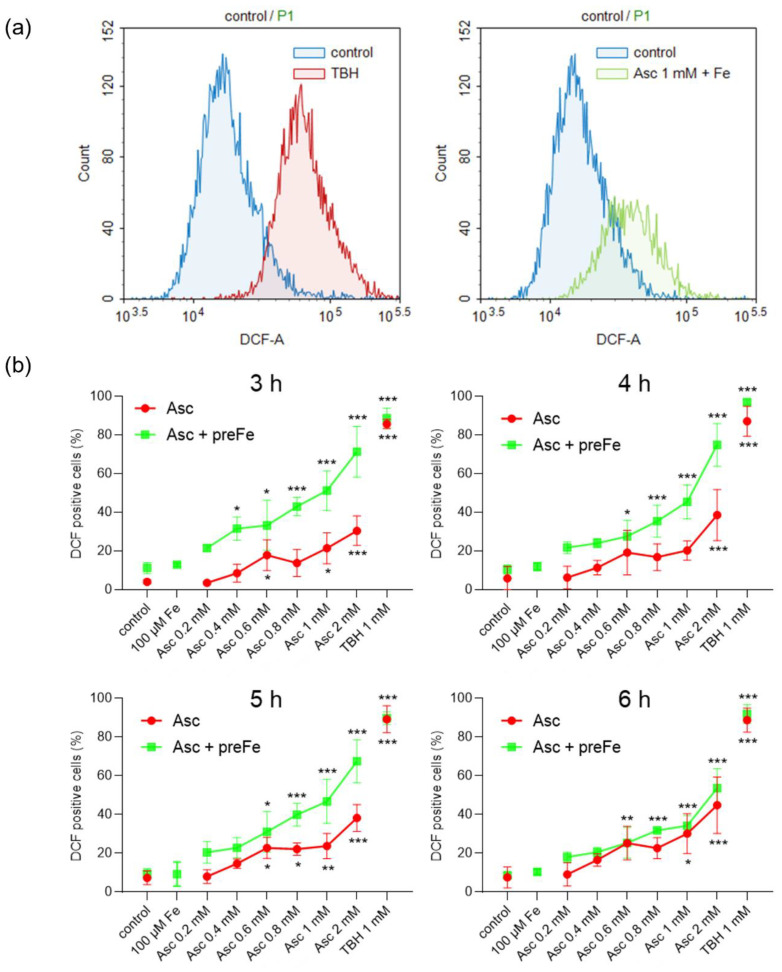
Effect of iron on ascorbate-induced ROS formation in human glioblastoma cells determined by DCFH-DA assay. SF268 cells were treated with different concentrations of Asc (0.2 mM, 0.4 mM, 0.6 mM, 0.8 mM, 1 mM, and 2 mM) for different time periods (3 h, 4 h, 5 h, and 6 h); 1 mM TBH served as a positive control. After Asc treatment, cells were stained with DCFH-DA and analyzed by flow cytometry (**a**). The DCF-A histograms were used to determine the percentages of DCF-positive cells, indicating cells with increased intracellular ROS levels (**b**). Cells were either treated with Asc alone (red dots) or pre-incubated with FeCl_3_ (100 µM) for 24 h immediately prior to Asc treatment (green squares). Three independent experiments were performed. Error bars represent mean ± SD, statistical analysis with one-way ANOVA and subsequent Dunnett’s multiple comparisons test, confidence interval 95%. *: *p* ≤ 0.05; **: *p* ≤ 0.01; ***: *p* ≤ 0.001. Asc, ascorbate; DCF, dichlorofluorescein; DCFH-DA, dichlorodihydrofluorescein diacetate; Fe, ferric chloride (FeCl_3_); pre, pre-incubation; ROS, reactive oxygen species; TBH, tert-butyl hydroperoxide.

To answer the question of whether pre-incubation with iron can also enhance ascorbate-induced ROS generation, intracellular ROS levels were examined after different treatment periods using the DCFH-DA assay. This allowed the detection of increased intracellular ROS generation by fluorescence intensity, as exemplified in Figure 3a for the positive control (TBH) and treatment with 1 mM ascorbate after pre-incubation with iron. The ROS levels in glioblastoma cells treated with ascorbate, as monotherapy and in combination with iron, were determined at four time points (3 h, 4 h, 5 h, and 6 h). In addition to the basal characteristics of ROS management within malignant entities, glioblastoma cells showed a significant dose-dependent ascorbate-induced increase in cellular ROS (Figure 3b, Supplementary Figures S9 and S10). Overall, for the ascorbate monotherapy and at all time points, a tendential dose-dependent increase in intracellular ROS levels was observed (Figure 3b; red lines). Consistent with the data shown in Figure 2, the increased cytotoxicity induced by pre-incubation with iron also appeared to lead to more ROS formation than with ascorbate alone (Figure 3b; green lines). The most pronounced effect of pre-incubation with iron on ROS induction was observed after 3 h of treatment. Here, a significant increase in intracellular ROS formation was already observed after treatment with 0.4 mM ascorbate, and at almost all concentrations, ROS levels were more than doubled compared to ascorbate treatment alone.

### 3.4. Ascorbate-Induced Cell Death Shows No Signs of Apoptosis

**Figure 4:**
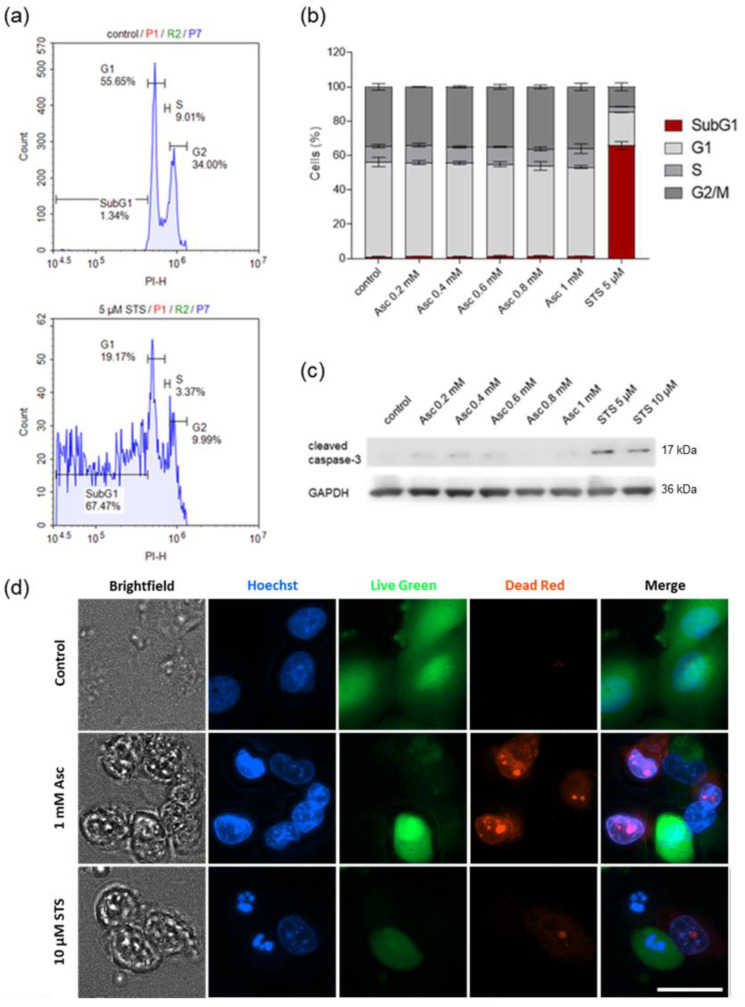
Cell cycle analysis and evaluation of apoptosis by high-dose ascorbate in human glioblastoma cells. SF268 cells were treated with different concentrations of Asc for 24 h (0.2 mM, 0.4 mM, 0.6 mM, 0.8 mM, and 1 mM) and cell cycle analysis was performed after PI staining by flow cytometry. The PI-H histogram was used to determine the percentage of cells in each cell cycle phase as well as apoptotic cells, as exemplified for untreated cells and the positive control (5 µM STS) (**a**). No increase in SubG1 fraction was detected following treatment with high-dose Asc (**b**). Western blot analysis of caspase-3 cleavage in cell lysates of SF268 cells treated with the indicated Asc concentrations or STS as positive control for 6 h (**c**). Equal protein loading was confirmed by GAPDH detection. After treatment for 6 h with 1 mM Asc, nuclei were stained with Hoechst 33342, live cells with Live Green, and dead cells with Dead Red. Cells were photographed with the Lionheart FX automated microscope (BioTek Instruments, Inc.) (**d**). The white bar is equivalent to 100 µm. 10 µM STS served as a positive control. Three independent experiments were performed. Error bars represent the mean ± SD. Asc, ascorbate; GAPDH, glyceraldehyde-3-phosphate dehydrogenase; PI, propidium iodide; STS, staurosporine.

Patterns of ascorbate-induced ROS-dependent cell death were then investigated by cell cycle analysis, immunoblotting, and examination of nuclear morphological changes. STS was used as a representative apoptosis inducer and thus as a positive control. The histogram of PI fluorescence intensity was used to determine the percentage of cells in each cell cycle phase and the percentage of cells with fragmented DNA, as exemplified for untreated cells and STS treatment (Figure 4a). Cell cycle analysis showed a typical increase in the SubG1 fraction after treatment of cells with STS, as would also be expected during apoptosis (Figure 4b). In contrast, treatment with ascorbate at concentrations up to 1 mM for 24 h did not result in an increase in the SubG1 fraction in any of the three cell lines examined. Similarly, no effect of pharmacological ascorbate concentrations was observed for the other cell cycle phases.

To confirm these results, caspase-3 cleavage was assessed in the cells after treatment to check for the induction of caspase-dependent apoptosis by high-dose ascorbate (Figure 4c). Consistent with our previous observations, no clear caspase-3 activation was observed by ascorbate concentrations up to 1 mM in SF268 cells after 6 h of treatment. In addition, cell death and morphological changes were examined by fluorescence imaging after treatment (Figure 4d). After treatment of cells with STS, the number of live cells decreased while the number of dead cells increased, and typical apoptotic nuclear morphological changes were observed compared to untreated cells by Hoechst staining. While an increased number of dead cells was also observed after treatment with 1 mM ascorbate, the nuclei were more similar to untreated cells than to cells treated with the apoptosis inducer STS. Results were confirmed with both SF295 and U251 cells (Supplementary Figures S11 and S12).

### 3.5. Ascorbate-Induced Cell Death Is Iron-Dependent and Shows Signs of Ferroptosis

**Figure 5:**
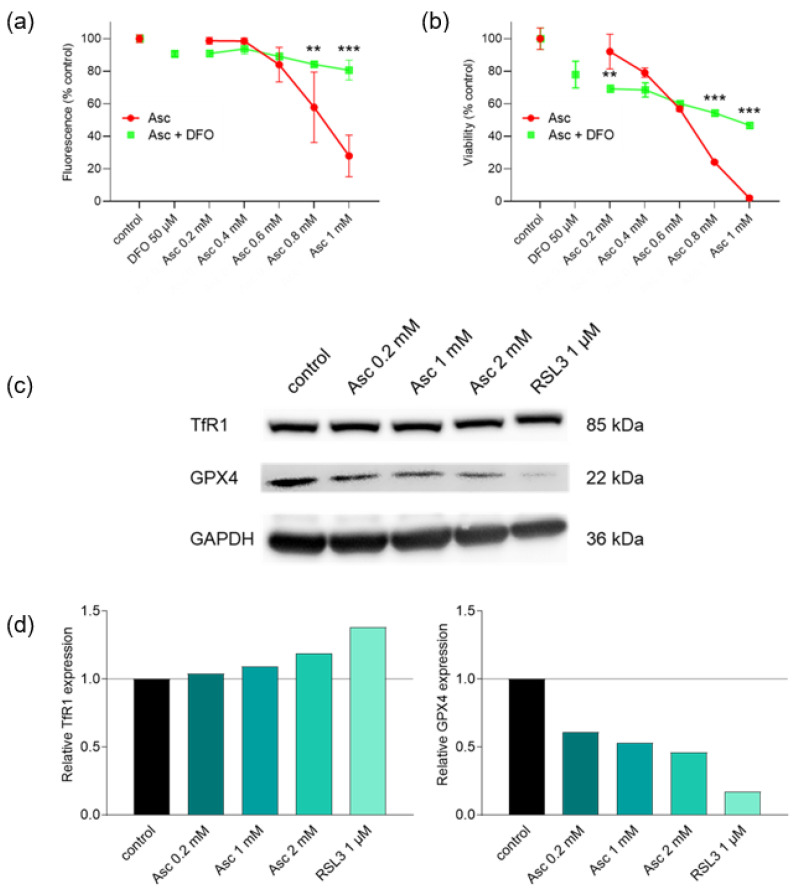
Investigation of ferroptosis by high-dose ascorbate in human glioblastoma cells. SF268 cells were treated with different concentrations of Asc for 24 h (0.2 mM, 0.4 mM, 0.6 mM, 0.8 mM, and 1 mM) with or without 50 µM DFO and cell viability was determined by MUH assay (**a**) and SRB assay (**b**). Western blot analysis of TfR1 and GPX4 expression in cell lysates of SF268 cells treated with the indicated Asc concentrations or RSL3 as a positive control for 8 h (**c**). Equal protein loading was confirmed by GAPDH detection. Protein expression was quantified by densitometry (ImageJ version 1.54, National Institutes of Health, Bethesda, MD, USA), normalized to GAPDH and shown relative to the untreated control (**d**). Error bars represent mean ± SD, statistical analysis with one-way ANOVA and subsequent Dunnett’s multiple comparisons test, confidence interval 95%. Asterisks indicate significant differences between ascorbate with and without DFO. **: *p* ≤ 0.01; ***: *p* ≤ 0.001. Asc, ascorbate; DFO, deferoxamine mesylate; GAPDH, glyceraldehyde-3-phosphate dehydrogenase; GPX4, glutathione peroxidase 4; RSL3, RAS-selective lethal 3; TfR1, transferrin receptor 1.

To investigate whether ferroptosis is involved in ascorbate-induced cell death, the influence of the ferroptosis inhibitor and iron chelator DFO on ascorbate treatment was examined (Figure 5a,b). DFO was able to almost abolish the cytotoxicity of high-dose ascorbate completely, which underlines the role of iron in the anti-tumor effect of ascorbate. In agreement with this, immunoblotting revealed an increase in TfR1 protein expression, which also indicates a ferroptotic cell death. Furthermore, it was found that GPX4 expression significantly decreased with increasing ascorbate concentrations, which confirms the assumption of ferroptosis as an ascorbate-induced cell death mechanism (Figure 5c). Densitometric quantification and normalization to GAPDH expression confirmed the changes in protein expression (Figure 5d). To further determine whether the formation of autophagosomes is involved in the ascorbate-induced cell death of glioblastoma cells, a Western blot analysis of LC3B expression was performed. However, there was no evidence of autophagy-related processes after treatment with high-dose ascorbate (Supplementary Figure S13).

## 4. Discussion

In the present work, the effects of high-dose ascorbate and DHA on glioblastoma cells were compared in terms of cytotoxicity, and a significant anti-proliferative effect was demonstrated with the use of high-dose ascorbate. We displayed that co-treatment with iron and ascorbate almost neutralized the toxicity induced by ascorbate in glioblastoma cells. In contrast, pre-incubation with ferric chloride followed by treatment with ascorbate was shown to exacerbate cytotoxicity. Irrespective of the treatment regimen, incubation with ascorbate and magnesium showed no significant advantage over ascorbate-induced toxicity alone in glioblastoma cells. Supporting the hypothesis of other studies [25], we demonstrated that high-dose ascorbate induced glioblastoma cell death without the typical signs of caspase-dependent apoptosis induction, such as an increased SubG1 fraction, DNA fragmentation, or cleaved caspase-3 fraction. Rather, the observed cell death indicated clear signs of iron dependence as well as increased TfR1 and decreased GPX4 protein expression, both of which are indicative of ferroptotic cell death [26,27].

There are data from colorectal cancer [28], non-small-cell lung cancer [4], and breast cancer cell lines [29] confirming that ascorbate displayed much higher cytotoxicity than DHA, but this information was lacking for glioblastoma cell lines. Since there is only one standard protocol for the treatment of glioblastoma and few other effective methods are available to date, understanding ascorbate-related cytotoxicity is of immense importance for the development of optimized treatment strategies. Despite the promising results regarding glioblastoma, high-dose ascorbate was also described to be effective against other brain tumors or tumors of the nervous system (examples of gliomas such as anaplastic astrocytoma, anaplastic oligoastrocytoma, anaplastic oligodendroglioma [30,31] (NCT02168270, and NCI-2016-00239), and also neuroblastoma [32] in vitro. For the medulloblastoma, which occurs most frequently in children and juveniles, there are unfortunately no data on high-dose ascorbate available. In vivo studies in rats demonstrated the retardation of glioblastoma growth and invasiveness after intravenous ascorbate injection without any detectable systemic adverse effects [33]. In humans, side effects of high-dose ascorbate, such as the development of kidney stones, as well as contraindications such as glucose-6-phosphate dehydrogenase deficiency, renal insufficiency, existing kidney stones, and hereditary iron storage disorders, are described [34]. Contraindications and possible adverse effects of high doses of iron (gastrointestinal complaints with oral intake, circulatory problems, and risk of iron poisoning with infusion) and magnesium (diarrhea) have to be taken into account as well [35,36,37]. Regarding tumor grade and (intracellular) ascorbate levels, a relationship between ascorbate and hypoxia-inducible factor-1 (HIF-1) was described. Low tumor tissue ascorbate levels were associated with strong activation of HIF-1 and ultimately tumor growth [38]. In breast cancer, a strong correlation between intracellular ascorbate, HIF-1 activation, and survival of the patients was demonstrated by Campbell et al. [39]. In addition, there was also a strong correlation between higher ascorbate levels and higher 5-hydroxymethylcytosine (5-hmC) in glioma tissue [40].

We investigated if the generated free radicals act as potent non-selective oxidants in secondary reactions by measuring the intracellular concentration of ROS after incubation with ascorbate in physiological to pharmacological concentrations. The results of Sinnberg et al. suggest that the generation of ROS from ascorbate is catalyzed by serum compounds and that severe hypoxia mediates resistance to ascorbate-induced inhibition of cell proliferation [41]. In addition to hypoxia, cobalt chloride [42,43], concomitant treatment with the detoxifying enzyme catalase [44,45], iron depletion or application of iron chelators [21], and the presence of extracellular proteins [22] can reduce ascorbate-associated extracellular ROS generation [46]. On the other hand, there are numerous approaches to enhance pharmacological ascorbate-mediated cytotoxicity and ROS generation in tumors. Co-treatments such as oxygenation, also known as hyperbaric oxygen treatment [41], addition of arsenic trioxide [47,48,49], or iron loading of the cell [19] potentiate tumor suppression. In addition, ferritin-caged copper nanoparticles (Fn-Cu) have been shown to enhance the therapeutic potential of ascorbate in cancer [50] and several copper-based anticancer strategies are under consideration [51]. Additionally, a significant number of preclinical and clinical studies have described the administration of high doses of ascorbate to enhance the effects of conventional cytotoxic therapies [1]. Among these combination treatments, there are approximately 30 clinical trials that therapeutically target the oxidative stress and ROS generation pathway, including different categories of drugs such as kinase inhibitors [52,53], kinase modulators [1], glycolysis inhibitors [54], as well as the cytotoxic effects of metal-based complexes [55]. Candidates that interfere with iron metabolism and promote specific iron-induced cell death play a prominent role in this ROS-driven treatment strategy [56]. In addition to the various agents targeting iron metabolism, we have shown that enrichment of tumor cells with ferric iron results in a very effective enhancement of ascorbate-induced cytotoxicity in glioblastoma cell lines. Thus, this approach of combining iron and high-dose ascorbate provides a biochemical basis for further research on selective killing of cancer cells by ROS-mediated mechanisms. In this context, the alteration of the cellular iron balance by iron supersaturation or depletion and the induction of iron-dependent phospholipid peroxidation has made ferroptosis a unique modality of cell death [56]. Since ferroptosis has been implicated in cancer, and so-called ‘therapy-persister’ cancer cells seem highly susceptible to ferroptosis inducers, understanding the regulatory mechanisms of this type of cell death is of immense importance [57].

Ferroptosis is a form of regulated cell death that is triggered by specific perturbations of the intracellular microenvironment, determined by the imbalance between iron accumulation, increased ROS production, and the antioxidant system that targets lipid peroxidation. Depending on the trigger, ferroptosis can also involve autophagic processes [56].

ROS have been identified as key molecules in several signaling pathways that regulate both cell survival and cell death (apoptosis) [58]. Oronowicz et al. demonstrated that ascorbate-induced ROS-mediated oxidative cytotoxicity in WERI-Rb1 cells is due to increased intracellular Ca^2+^ influx mediated by Gi/o-coupled G protein-coupled receptor (GPCR) via transient receptor potential channels (TRPs) [59]. In the context of ischemic brain stroke, electrophysiological patch clamp experiments demonstrated that vitamin C increases the whole-cell current of the large-conductance Ca^2+^-activated K^+^ channel (BK_Ca_). In this context, vitamin C increased the opening probability of the channel without changing its amplitude [60]. Autophagy, for its part, is a self-digestion process that degrades intracellular structures in response to stress, such as nutrient deprivation, and is involved in both cell survival and cell death. ROS can trigger autophagy via several mechanisms involving autophagy-related protease 4 (Atg4), catalase, and the mitochondrial electron transport chain, leading to both cell survival and death, which may be selective for cancer cells [58]. Ren et al. showed that radical-induced apoptosis in HeLa cells is independent of caspase-3, but is associated with a decrease of the mitochondrial transmembrane potential and can be characterized by the morphological and biochemical changes typical for apoptosis [61]. This caspase-independent apoptosis may become dominant at higher concentrations of ascorbate, mostly described in older studies [41,62]. Recent studies focus more on the exact mechanism and suggest that ferroptosis and autophagy-induced cell death occur independently, but both are mediated by iron-dependent ROS generation in breast cancer cells [63]. The observation that pharmacological ascorbate-induced cell death is iron-dependent, ROS-mediated, caspase-independent [56], and possibly involves autophagy [62,64] confirms that this cancer cell death has a ferroptotic component [25], which is also indicated by the results of this work, although in the case of the glioblastoma cells examined, ferroptosis-like cell death without clear signs of autophagy appears to be present. These studies imply that the mode of cell death may change with increasing ascorbate concentration.

A major finding of our study is the potentiation of ascorbate-induced toxicity by pre-incubation of glioblastoma cells with ferric chloride. Therefore, the combination with iron could be a promising approach for the treatment of glioblastoma, which could be easily transferred to a clinical setting. However, since elevation of iron levels in tumor cells has seemingly favorable effects in this context while elevation of serum iron levels during high-dose ascorbate administration may have unfavorable effects on treatment efficacy, special attention should be paid to the timing of iron application and the half-life of iron preparations.

## 5. Conclusions

High-dose ascorbate, but not DHA, induces severe cytotoxicity in human glioblastoma cell lines. Ascorbate-induced ROS formation can be significantly increased and accelerated by pre-treatment of glioblastoma cells with ferric iron prior to ascorbate treatment by increasing intracellular labile iron, resulting in augmented cell death. This promising combination therapy could increase the efficacy of high-dose ascorbate treatment in glioblastoma patients.

## 6. Innovation

High-dose ascorbate in combination with radiotherapy and temozolomide has demonstrated selective toxicity, tolerability, and potential efficacy in a Phase I trial in glioblastoma patients. In the current work, we demonstrate for the first time the potentiation of the selective toxicity of high-dose ascorbate by pre-incubating glioblastoma cells with iron and the enhancement of ROS generation to reduce tumor cell growth. Our in vitro results thus contribute to the expansion of treatment options for glioblastoma as a new approach for the management of this extremely aggressive tumor entity with very poor prognosis.

## Data Availability

Data is contained within the article and Supplementary Materials.

## Supplementary Materials

The following supporting information can be downloaded at: https://www.mdpi.com/article/10.3390/antiox13091095/s1, Figure S1: Evaluation of the sensitivity of SF295 cells to high-dose ascorbate and DHA treatment; Figure S2: Evaluation of the sensitivity of U251 cells to high-dose ascorbate and DHA treatment; Figure S3: Evaluation of the effect of high-dose ascorbate treatment on the viability of human non-malignant cell lines; Figure S4: Effect of iron and magnesium on ascorbate-induced cytotoxicity in SF295 cells shown by MUH assay; Figure S5: Effect of iron and magnesium on ascorbate-induced cytotoxicity in U251 cells shown by MUH assay; Figure S6: Effect of iron and magnesium on ascorbate-induced cytotoxicity in SF268 cells shown by SRB assay; Figure S7: Effect of iron and magnesium on ascorbate-induced cytotoxicity in SF295 cells shown by SRB assay; Figure S8: Effect of iron and magnesium on ascorbate-induced cytotoxicity in U251 cells shown by SRB assay; Figure S9: Effect of iron on ascorbate-induced ROS formation in SF295 cells determined by DCFH-DA assay; Figure S10: Effect of iron on ascorbate-induced ROS formation in U251 cells determined by DCFH-DA assay; Figure S11: Cell cycle analysis and evaluation of apoptosis by high-dose ascorbate in SF295 cells; Figure S12: Cell cycle analysis and evaluation of apoptosis by high-dose ascorbate in U251 cells; Figure S13: Western blot analysis of LC3B-II in cell lysates of SF268 cells treated with the indicated Asc concentrations or CQ and RAPA as a positive control for 8 h.

## Abbreviations

Atg4	autophagy-related protease 4
BBB	blood-brain-barrier
BK_Ca_	large-conductance Ca^2+^-activated K^+^ channel
BSA	bovine serum albumin
CQ	chloroquine
DHA	dehydroascorbic acid
DFO	deferoxamine mesylate
DCFH-DA	dichlorodihydrofluorescein diacetate
FCS	fetal calf serum
Fe	FeCl_3_
Fe^2+^	ferrous iron
Fe^3+^	ferric iron
GAPDH	glyceraldehyde-3-phosphate dehydrogenase
GPCR	G protein-coupled receptor
GPX4	glutathione peroxidase 4
HIF-1	hypoxia-inducible factor-1
5-hmC	5-hydroxymethylcytosine
HRP	horseradish peroxidase
LC3B	microtubule-associated proteins 1A/1B light chain 3B
LIP	labile iron pool
Mg	magnesium
MUH	4-methylumbelliferyl heptanoate
NSCLC	non-small cell lung cancer
OS	overall survival
PI	propidium iodide
PMSF	phenylmethanesulfonyl fluoride
PVDF	polyvinylidene difluoride
RAPA	rapamycin
ROS	reactive oxygen species
RSL3	RAS-selective lethal 3
SDS	sodium dodecyl sulfate
SRB	sulforhodamine B
STS	staurosporine
SVCT2	sodium-dependent vitamin C transporter 2
TBH	tert-butyl hydroperoxide
TBST	Tris-buffered saline with Tween-20
TCA	trichloroacetic acid
TfR1	transferrin receptor 1
TMZ	temozolomide
TRP	transient receptor potential channel
