# Supplementary material for: Analysis of High-Dose Ascorbate-Induced Cytotoxicity in Human Glioblastoma Cells and the Role of Dehydroascorbic Acid and Iron
Source: Antioxidants (Basel). 2024 Sep 10;13(9):1095. doi: 10.3390/antiox13091095 (PMC11429401; doi:10.3390/antiox13091095)
Supplement: Supplementary file 1 [file antioxidants-13-01095-s001.zip › antioxidants-3149596-supplementary.pdf]

Supplementary material:

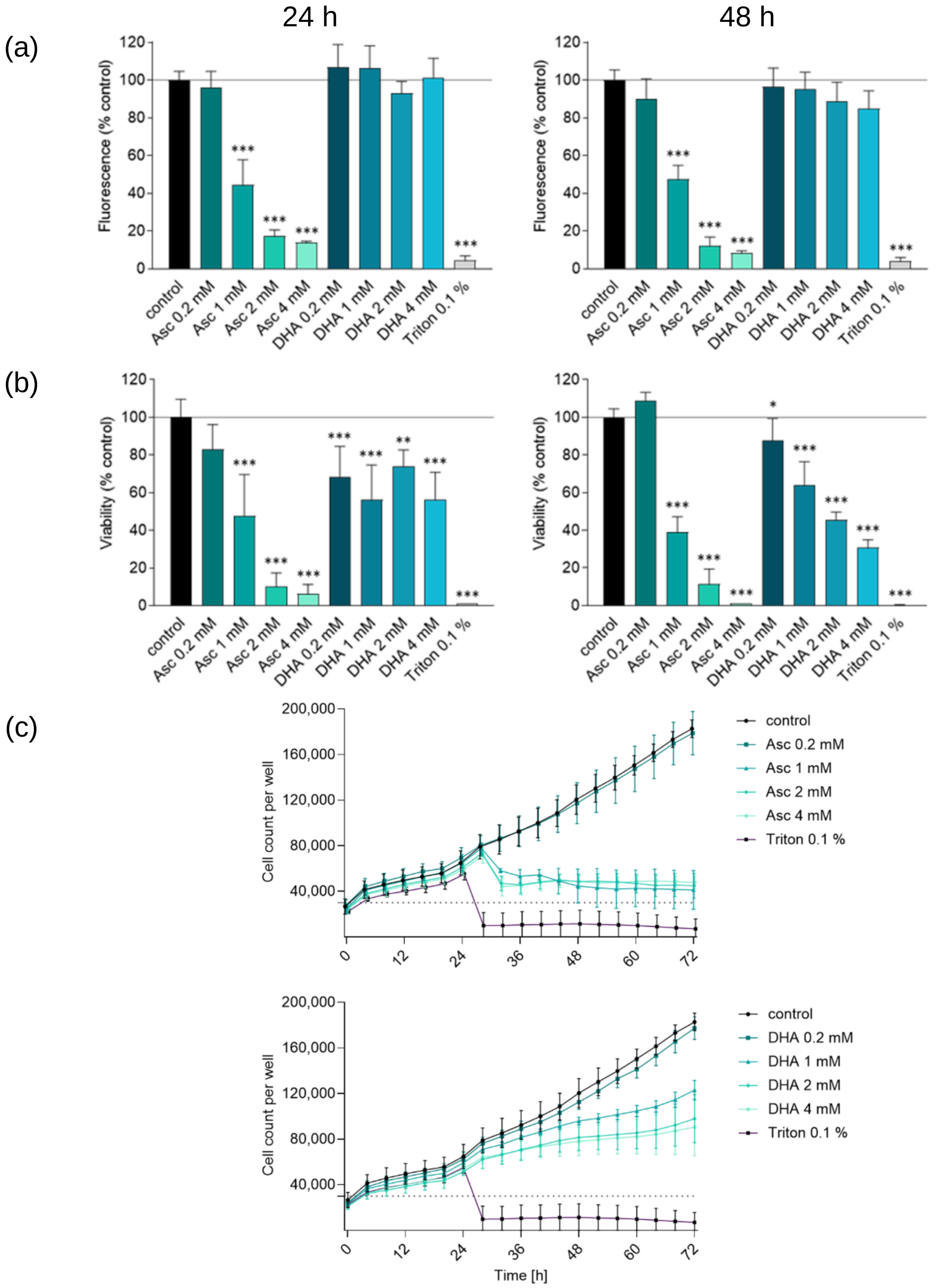

Figure S1: Evaluation of the sensitivity of SF295 cells to high-dose ascorbate and DHA treatment. Cells were treated for 24 h or 48 h with different concentrations of Asc or DHA (0.2 mM, 1 mM, 2 mM, and 4 mM). Triton X-100 at 0.1% (v/v) served as a positive control. Cell viability was assessed by MUH assay (**a**) and SRB assay (**b**). Results are presented as percentage of fluorescence intensity and viability, respectively, compared to the untreated control. (**c**) Anti-proliferative effects were detected by cell number determination using the Lionheart FX automated microscope (BioTek Instruments, Inc., Winooski, USA). Treatment was performed 24 h after seeding. Results are presented as cell number per well every 4 h. Three independent experiments were performed, each in duplicates. Error bars represent mean ± SD, statistical analysis with one-way ANOVA and subsequent Dunnett's multiple comparisons test,

confidence interval 95%. *: $p \leq 0.05$; **: $p \leq 0.01$; ***: $p \leq 0.001$. Asc, ascorbate; DHA, dehydroascorbic acid; MUH, 4-methylumbelliferyl heptanoate; SRB, sulforhodamine B.

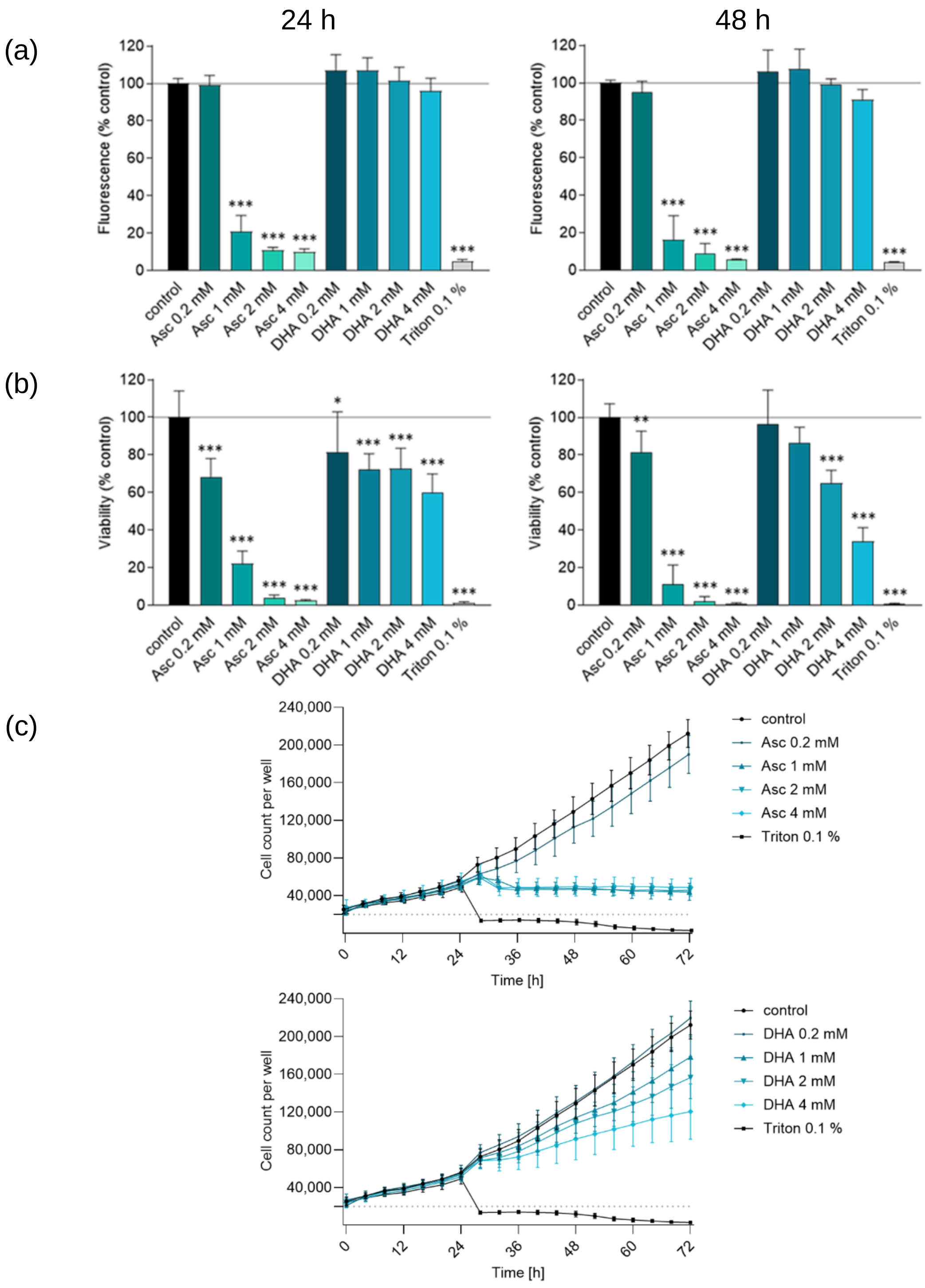

Figure S2: Evaluation of the sensitivity of U251 cells to high-dose ascorbate and DHA treatment. Cells were treated for 24 h or 48 h with different concentrations of Asc or DHA (0.2 mM, 1 mM, 2 mM, and 4 mM). Triton X-100 at 0.1% (v/v) served as a positive control. Cell viability was assessed by MUH assay (**a**) and SRB assay (**b**). Results are presented as percentage of fluorescence intensity and viability, respectively, compared to the untreated control. (**c**) Anti-proliferative effects were detected by cell number determination using the Lionheart FX automated microscope (BioTek Instruments, Inc., Winooski, USA). Treatment was performed 24 h after seeding. Results are presented as cell number per well every 4 h. Three independent experiments were performed, each in duplicates. Error bars represent mean ± SD, statistical analysis with one-way ANOVA and subsequent Dunnett's multiple comparisons test, confidence interval 95%. *: $p \le 0.05$; **: $p \le 0.01$; ***: $p \le 0.001$. Asc, ascorbate; DHA, dehydroascorbic acid; MUH, 4-methylumbelliferyl heptanoate; SRB, sulforhodamine B.

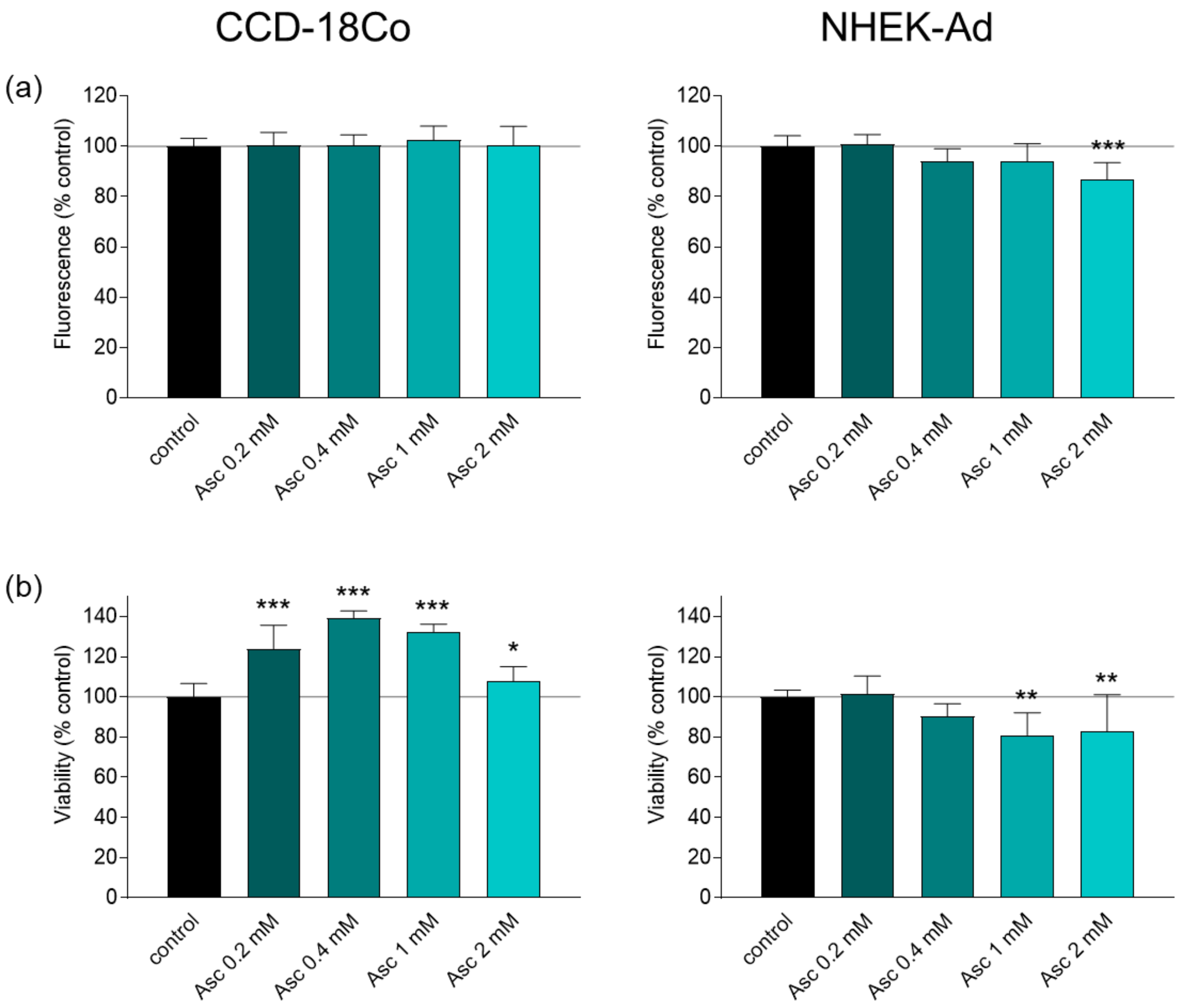

Figure S3: Evaluation of the effect of high-dose ascorbate treatment on the viability of human non-malignant cell lines. CCD-18Co and NHEK-Ad cells were treated for 72 h with different ascorbate concentrations (0.2 mM, 0.4 mM, 1 mM, and 2 mM). Cell viability was assessed by MUH assay (**a**) and SRB assay (**b**). Results are presented as percentage of fluorescence intensity and viability, respectively, compared to the untreated control. Treatment was performed 24 h after seeding. Three independent experiments were performed, each in triplicates. Error bars represent mean ± SD, statistical analysis with one-way ANOVA and subsequent Dunnett's multiple comparisons test, confidence interval 95%. *: $p \leq 0.05$; **: $p \leq 0.01$; ***: $p \leq 0.001$. Asc, ascorbate; MUH, 4-methylumbelliferyl heptanoate; SRB, sulforhodamine B.

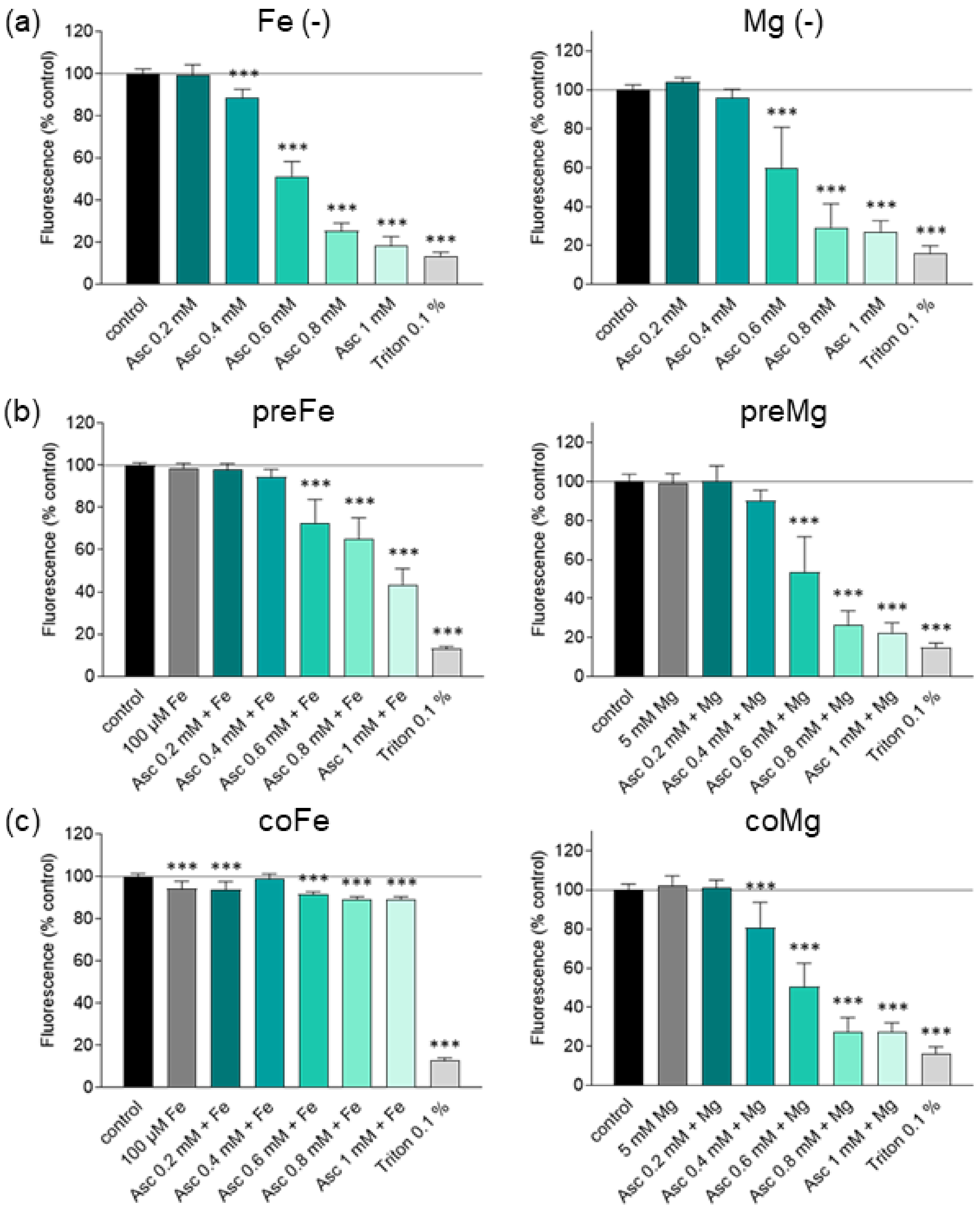

Figure S4: Effect of iron and magnesium on ascorbate-induced cytotoxicity in SF295 cells shown by MUH assay. Cells were treated with different concentrations of Asc for 24 h (0.2 mM, 0.4 mM, 0.6 mM, 0.8 mM, and 1 mM). Triton X-100 at 0.1% (v/v) served as a positive control. Cells were either treated with Asc alone (**a**), pre-incubated with $FeCl_3$ (100 µM) or $MgCl_2$ (5 mM) for 24 h immediately prior to Asc treatment (**b**), or co-incubated with Asc and $FeCl_3$ or $MgCl_2$ (**c**). Results are presented as percentage of fluorescence intensity compared to the untreated control. Three independent experiments were performed, each in duplicates. Error bars represent mean ± SD, statistical analysis with one-way ANOVA and subsequent Dunnett's multiple comparisons test, confidence interval 95%. ***: $p \leq 0.001$. Asc, ascorbate; co, co-incubation; Fe, ferric chloride ($FeCl_3$); Mg, magnesium chloride ($MgCl_2$); MUH, 4-methylumbelliferyl heptanoate; pre, pre-incubation.

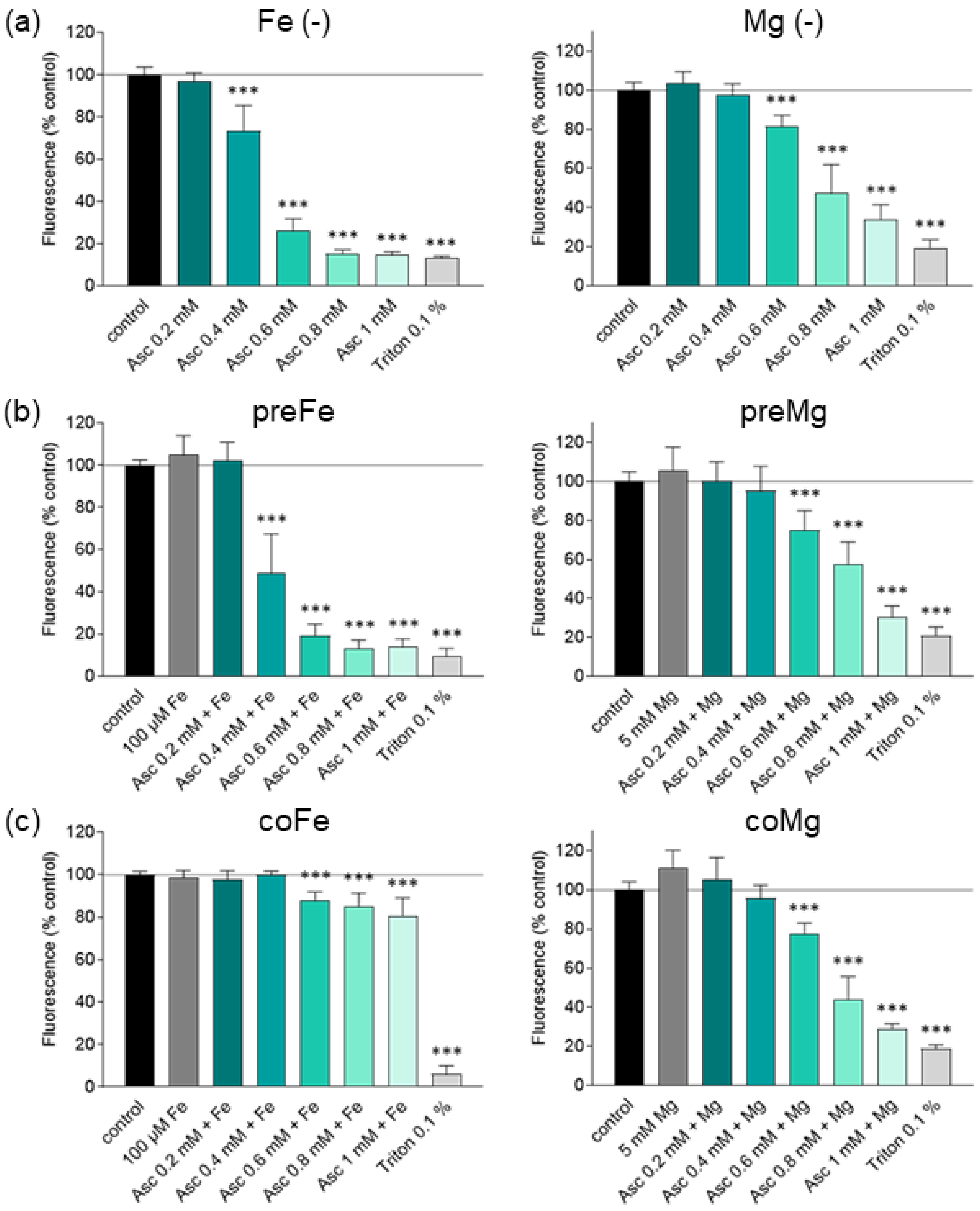

Figure S5: Effect of iron and magnesium on ascorbate-induced cytotoxicity in U251 cells shown by MUH assay. Cells were treated with different concentrations of Asc for 24 h (0.2 mM, 0.4 mM, 0.6 mM, 0.8 mM, and 1 mM). Triton X-100 at 0.1% (v/v) served as a positive control. Cells were either treated with Asc alone (**a**), pre-incubated with $FeCl_3$ (100 µM) or $MgCl_2$ (5 mM) for 24 h immediately prior to Asc treatment (**b**), or co-incubated with Asc and $FeCl_3$ or $MgCl_2$ (**c**). Results are presented as percentage of fluorescence intensity compared to the untreated control. Three independent experiments were performed, each in duplicates. Error bars represent mean ± SD, statistical analysis with one-way ANOVA and subsequent Dunnett's multiple comparisons test, confidence interval 95%. ***: $p \leq 0.001$. Asc, ascorbate; co, co-incubation; Fe, ferric chloride ($FeCl_3$); Mg, magnesium chloride ($MgCl_2$); MUH, 4-methylumbelliferyl heptanoate; pre, pre-incubation.

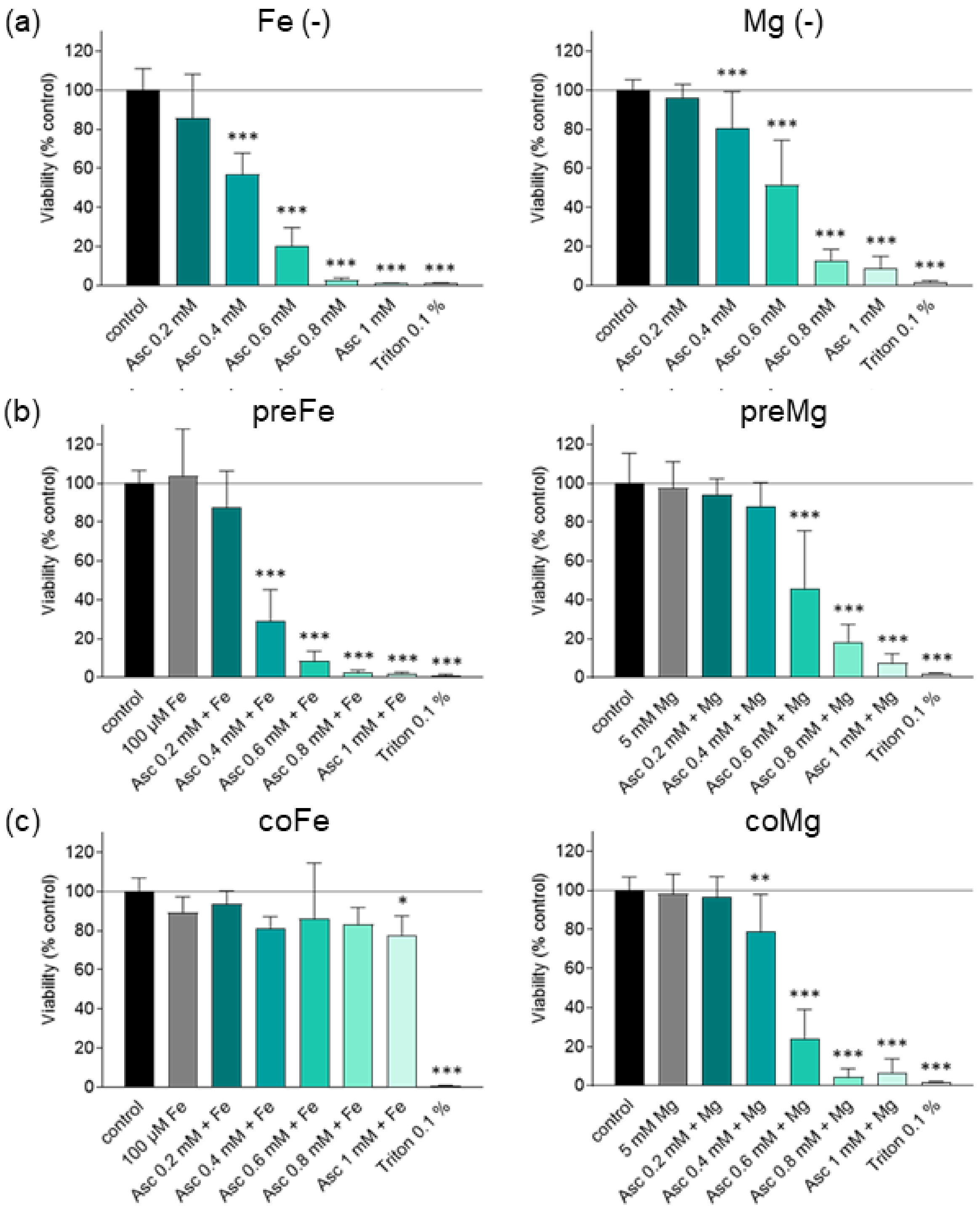

Figure S6: Effect of iron and magnesium on ascorbate-induced cytotoxicity in SF268 cells shown by SRB assay. Cells were treated with different concentrations of Asc for 24 h (0.2 mM, 0.4 mM, 0.6 mM, 0.8 mM, and 1 mM). Triton X-100 at 0.1% (v/v) served as a positive control. Cells were either treated with Asc alone (**a**), pre-incubated with $FeCl_3$ (100 µM) or $MgCl_2$ (5 mM) for 24 h immediately prior to Asc treatment (**b**), or co-incubated with Asc and $FeCl_3$ or $MgCl_2$ (**c**). Results are presented as percentage of fluorescence intensity compared to the untreated control. Three independent experiments were performed, each in duplicates. Error bars represent mean ± SD, statistical analysis with one-way ANOVA and subsequent Dunnett's multiple comparisons test, confidence interval 95%. *: $p \leq 0.05$; **: $p \leq 0.01$; ***: $p \leq 0.001$. Asc, ascorbate; co, co-incubation; Fe, ferric chloride ($FeCl_3$); Mg, magnesium chloride ($MgCl_2$); pre, pre-incubation; SRB, sulforhodamine B.

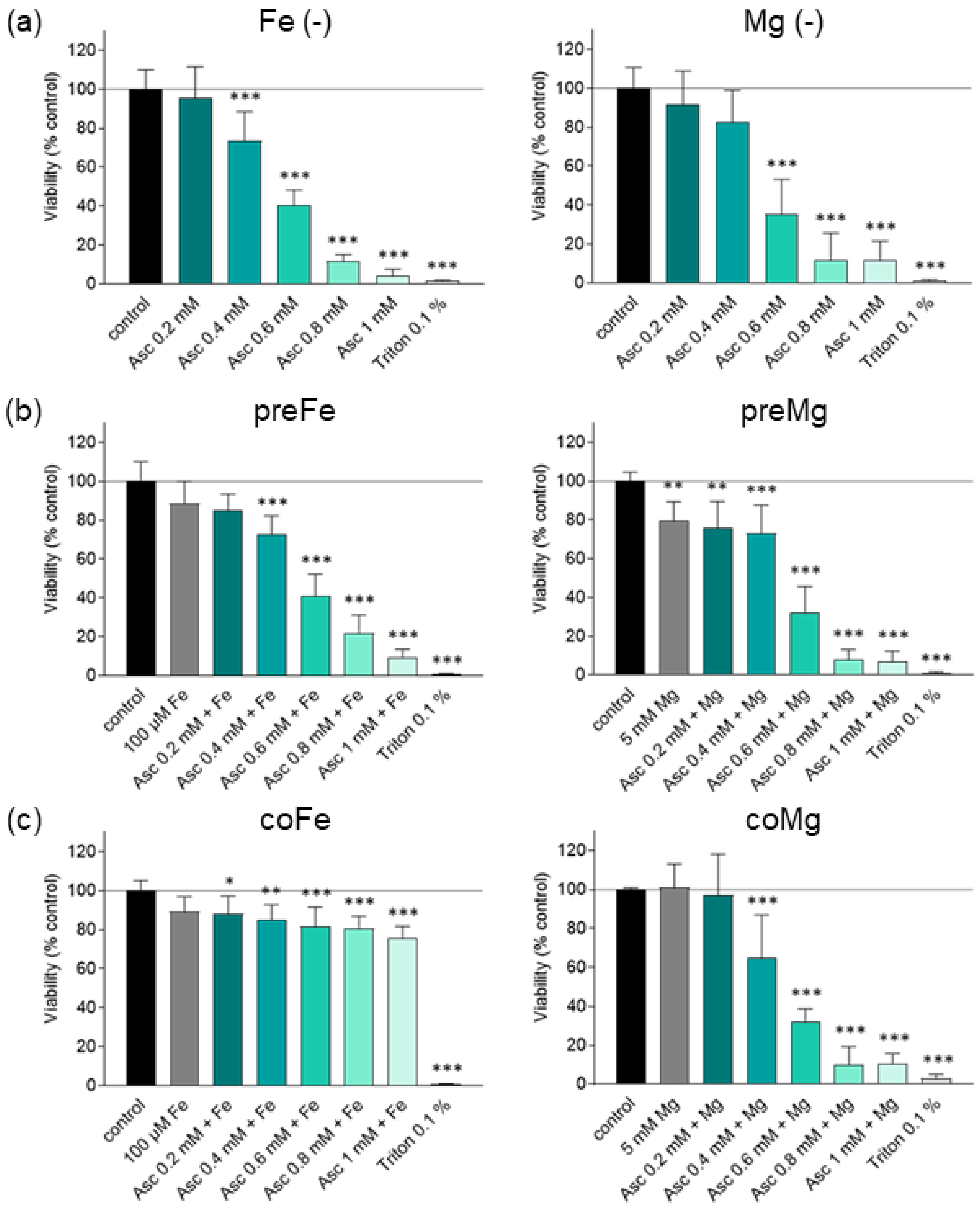

Figure S7: Effect of iron and magnesium on ascorbate-induced cytotoxicity in SF295 cells shown by SRB assay. Cells were treated with different concentrations of Asc for 24 h (0.2 mM, 0.4 mM, 0.6 mM, 0.8 mM, and 1 mM). Triton X-100 at 0.1% (v/v) served as a positive control. Cells were either treated with Asc alone (**a**), pre-incubated with $FeCl_3$ (100 µM) or $MgCl_2$ (5 mM) for 24 h immediately prior to Asc treatment (**b**), or co-incubated with Asc and $FeCl_3$ or $MgCl_2$ (**c**). Results are presented as percentage of fluorescence intensity compared to the untreated control. Three independent experiments were performed, each in duplicates. Error bars represent mean ± SD, statistical analysis with one-way ANOVA and subsequent Dunnett's multiple comparisons test, confidence interval 95%. *: $p \leq 0.05$; **: $p \leq 0.01$; ***: $p \leq 0.001$. Asc, ascorbate; co, co-incubation; Fe, ferric chloride ($FeCl_3$); Mg, magnesium chloride ($MgCl_2$); pre, pre-incubation; SRB, sulforhodamine B.

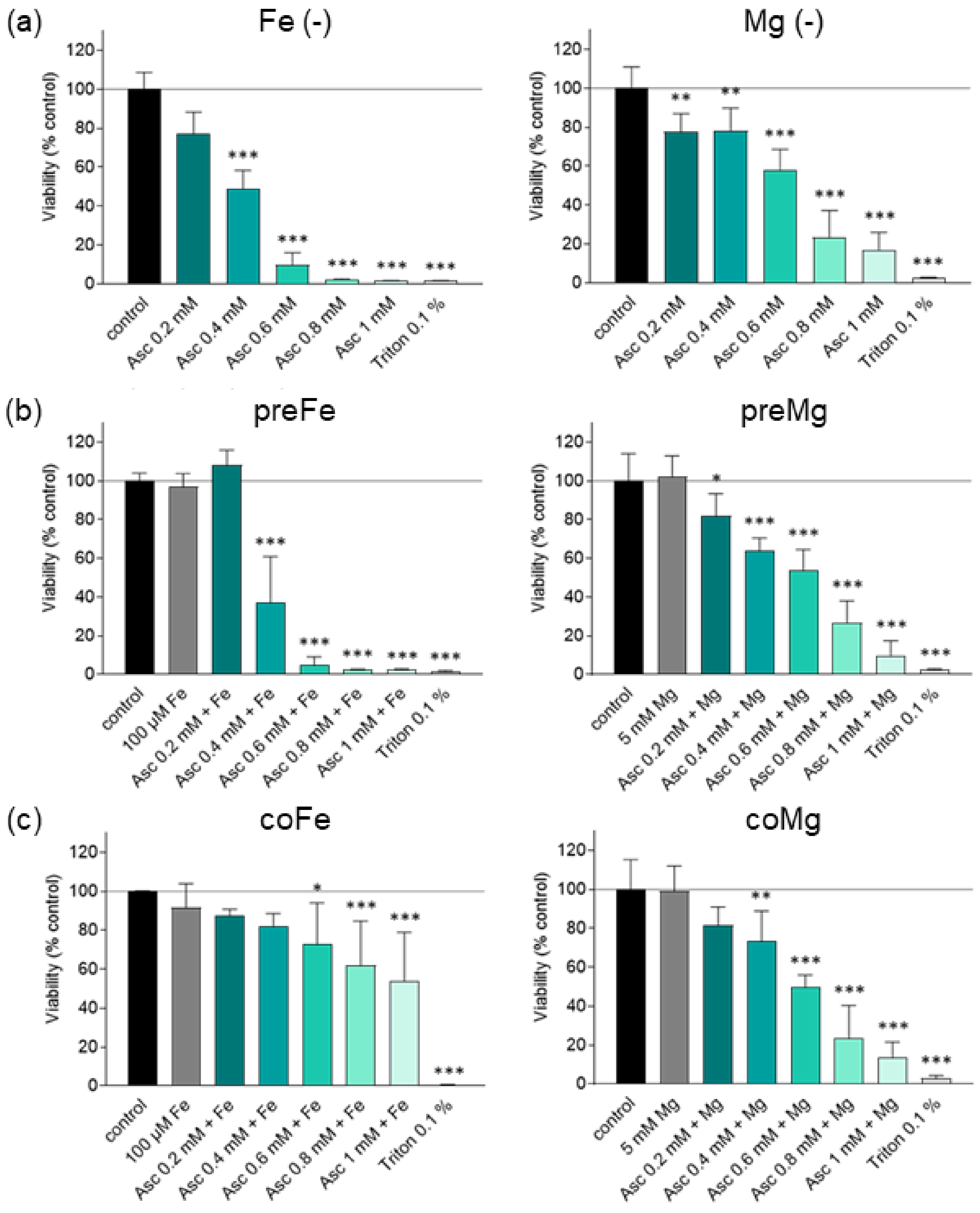

Figure S8: Effect of iron and magnesium on ascorbate-induced cytotoxicity in U251 cells shown by SRB assay. Cells were treated with different concentrations of Asc for 24 h (0.2 mM, 0.4 mM, 0.6 mM, 0.8 mM, and 1 mM). Triton X-100 at 0.1% (v/v) served as a positive control. Cells were either treated with Asc alone (**a**), pre-incubated with $FeCl_3$ (100 µM) or $MgCl_2$ (5 mM) for 24 h immediately prior to Asc treatment (**b**), or co-incubated with Asc and $FeCl_3$ or $MgCl_2$ (**c**). Results are presented as percentage of fluorescence intensity compared to the untreated control. Three independent experiments were performed, each in duplicates. Error bars represent mean ± SD, statistical analysis with one-way ANOVA and subsequent Dunnett's multiple comparisons test, confidence interval 95%. *: $p \leq 0.05$; **: $p \leq 0.01$; ***: $p \leq 0.001$. Asc, ascorbate; co, co-incubation; Fe, ferric chloride ($FeCl_3$); Mg, magnesium chloride ($MgCl_2$); pre, pre-incubation; SRB, sulforhodamine B.

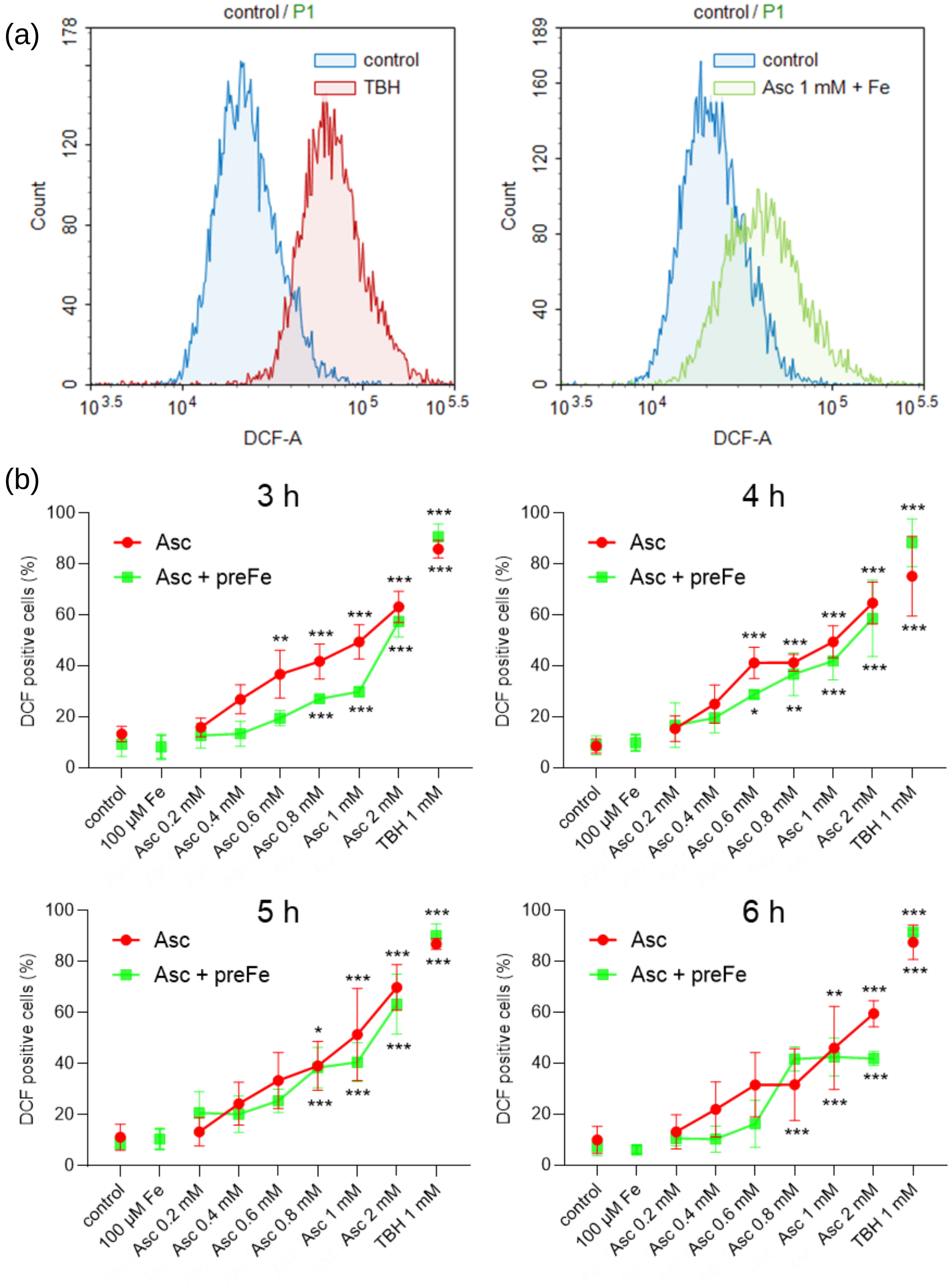

Figure S9: Effect of iron on ascorbate-induced ROS formation in SF295 cells determined by DCFH-DA assay. Cells were treated with different concentrations of Asc (0.2 mM, 0.4 mM, 0.6 mM, 0.8 mM, 1 mM, and 2 mM) for different time periods (3 h, 4 h, 5 h, and 6 h). 1 mM TBH served as a positive control. After Asc treatment, cells were stained with DCFH-DA and analyzed by flow cytometry (**a**). The DCF-A histograms were used to determine the percentages of DCF-positive cells, indicating cells with increased intracellular ROS levels (**b**). Cells were either treated with Asc alone (red dots) or pre-incubated with $FeCl_3$ (100 µM) for 24 h immediately prior to Asc treatment (green squares). Three independent experiments were performed. Error bars represent mean ± SD, statistical analysis with one-way ANOVA and subsequent Dunnett's multiple comparisons test, confidence interval 95%. *: $p \leq 0.05$; **: $p \leq 0.01$; ***: $p \leq 0.001$. Asc, ascorbate; DCF, dichlorofluorescein; DCFH-DA, dichlorodihydrofluorescein diacetate; Fe, ferric chloride ($FeCl_3$); pre, pre-incubation; ROS, reactive oxygen species; TBH, tert-butyl hydroperoxide.

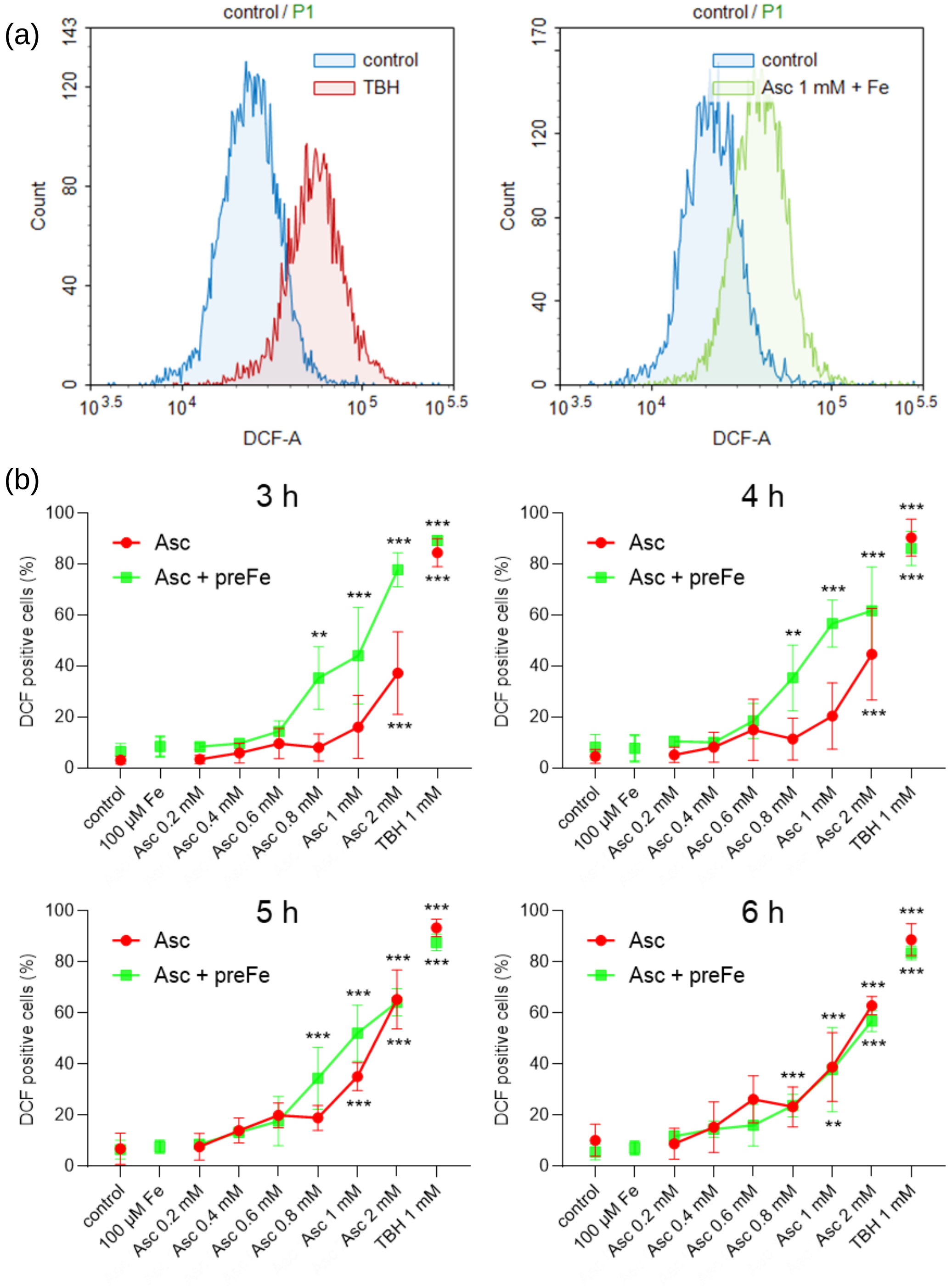

Figure S10: Effect of iron on ascorbate-induced ROS formation in U251 cells determined by DCFH-DA assay. Cells were treated with different concentrations of Asc (0.2 mM, 0.4 mM, 0.6 mM, 0.8 mM, 1 mM, and 2 mM) for different time periods (3 h, 4 h, 5 h, and 6 h). 1 mM TBH served as a positive control. After Asc treatment, cells were stained with DCFH-DA and analyzed by flow cytometry (**a**). The DCF-A histograms were used to determine the percentages of DCF-positive cells, indicating cells with increased intracellular ROS levels (**b**). Cells were either treated with Asc alone (red dots) or pre-incubated with $FeCl_3$ (100 µM) for 24 h immediately prior to Asc treatment (green squares). Three independent experiments were performed. Error bars represent mean ± SD, statistical analysis with one-way ANOVA and subsequent Dunnett's multiple comparisons test, confidence interval 95%. **: $p \leq 0.01$; ***: $p \leq 0.001$. Asc, ascorbate; DCF, dichlorofluorescein; DCFH-DA, dichlorodihydrofluorescein diacetate; Fe, ferric chloride ($FeCl_3$); pre, pre-incubation; ROS, reactive oxygen species; TBH, tert-butyl hydroperoxide.

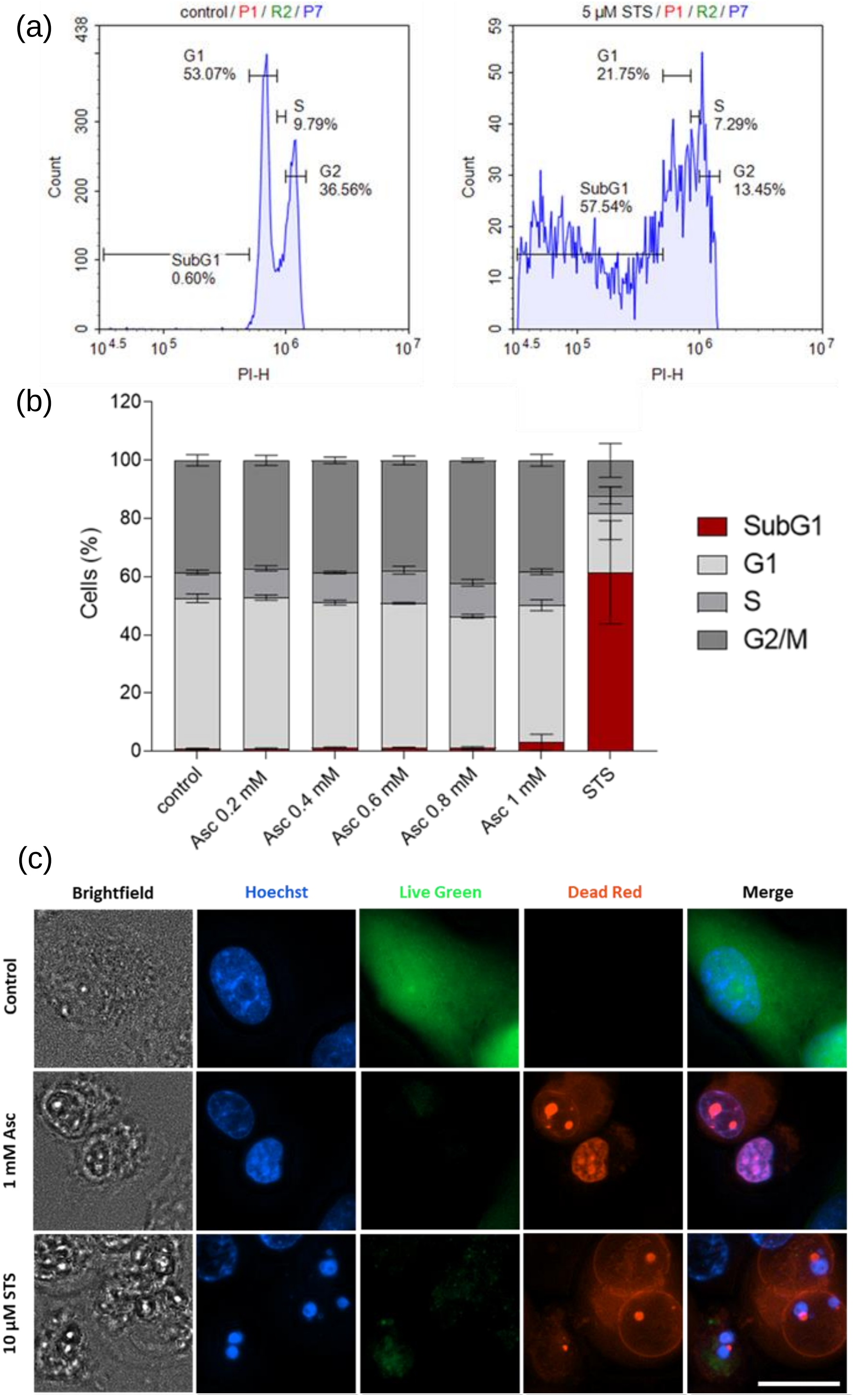

Figure S11: Cell cycle analysis and evaluation of apoptosis by high-dose ascorbate in SF295 cells. Cells were treated with different concentrations of Asc for 24 h (0.2 mM, 0.4 mM, 0.6 mM, 0.8 mM, and 1 mM) and cell cycle analysis was performed after PI staining by flow cytometry. The PI-H histogram was used to determine the percentage of cells in each cell cycle phase as well as apoptotic cells, as exemplified for untreated cells and the positive control (5 µM STS) (**a**). No increase in SubG1 fraction was detected following treatment with high-dose Asc (**b**). After treatment for 6 h with 1 mM Asc, nuclei were stained with Hoechst 33342, live cells with Live Green, and dead cells with Dead Red. Cells were photographed with the Lionheart FX automated microscope (BioTek Instruments, Inc.) (**c**). The white bar is equivalent to 25 µm. 10 µM STS served as a positive control. Three independent experiments were performed. Error bars represent the mean ± SD. Asc, ascorbate; PI, propidium iodide; STS, staurosporine.

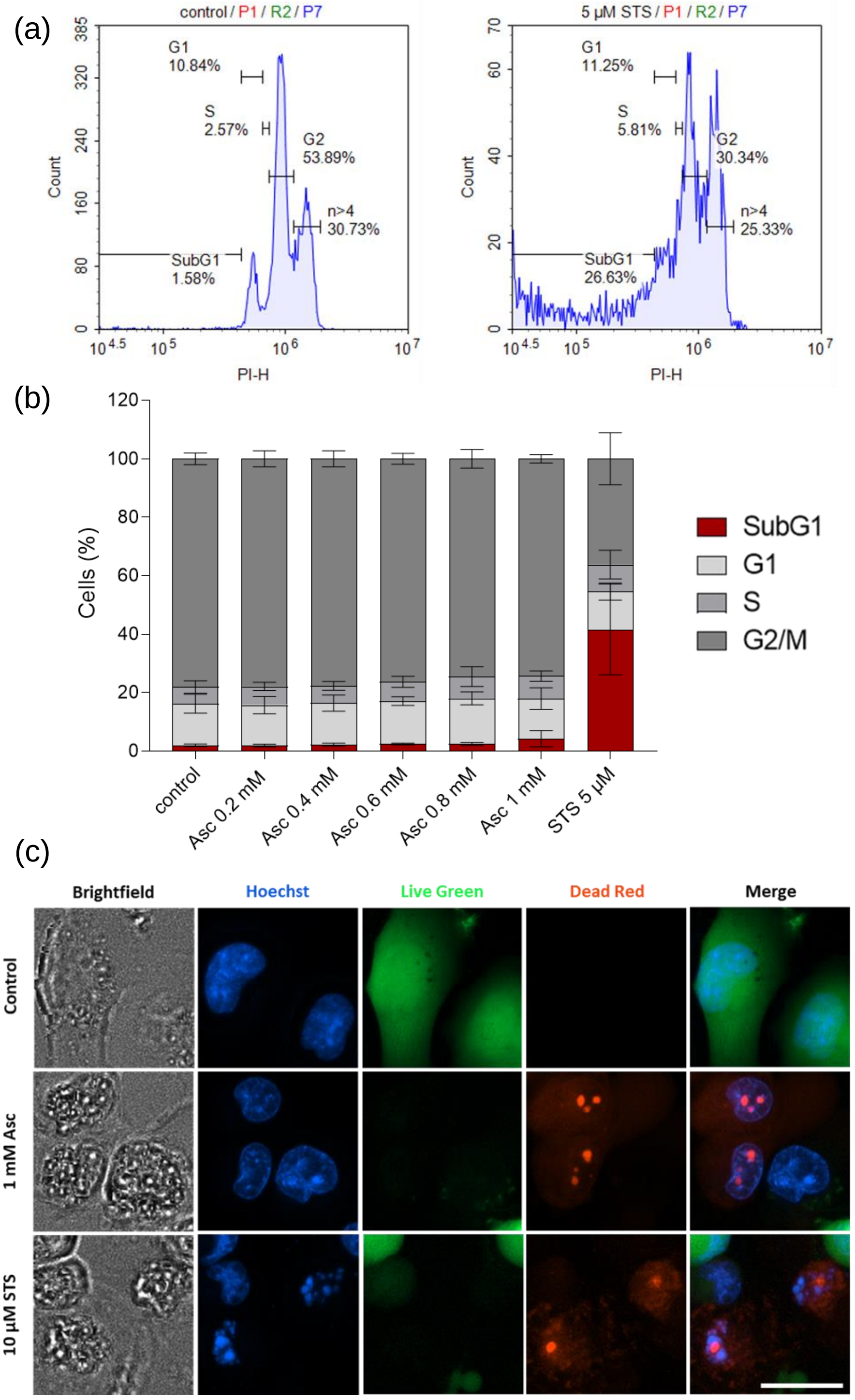

Figure S12: Cell cycle analysis and evaluation of apoptosis by high-dose ascorbate in U251 cells. Cells were treated with different concentrations of Asc for 24 h (0.2 mM, 0.4 mM, 0.6 mM, 0.8 mM, and 1 mM) and cell cycle analysis was performed after PI staining by flow cytometry. The PI-H histogram was used to determine the percentage of cells in each cell cycle phase as well as apoptotic cells, as exemplified for untreated cells and the positive control (5 μM STS) (**a**). No increase in SubG1 fraction was detected following treatment with high-dose Asc (**b**). After treatment for 6 h with 1 mM Asc, nuclei were stained with Hoechst 33342, live cells with Live Green, and dead cells with Dead Red. Cells were photographed with the Lionheart FX automated microscope (BioTek Instruments, Inc.) (**c**). The white bar is equivalent to 25 μm. 10 μM STS served as a positive control. Three independent experiments were performed. Error bars represent the mean ± SD. Asc, ascorbate; PI, propidium iodide; STS, staurosporine.

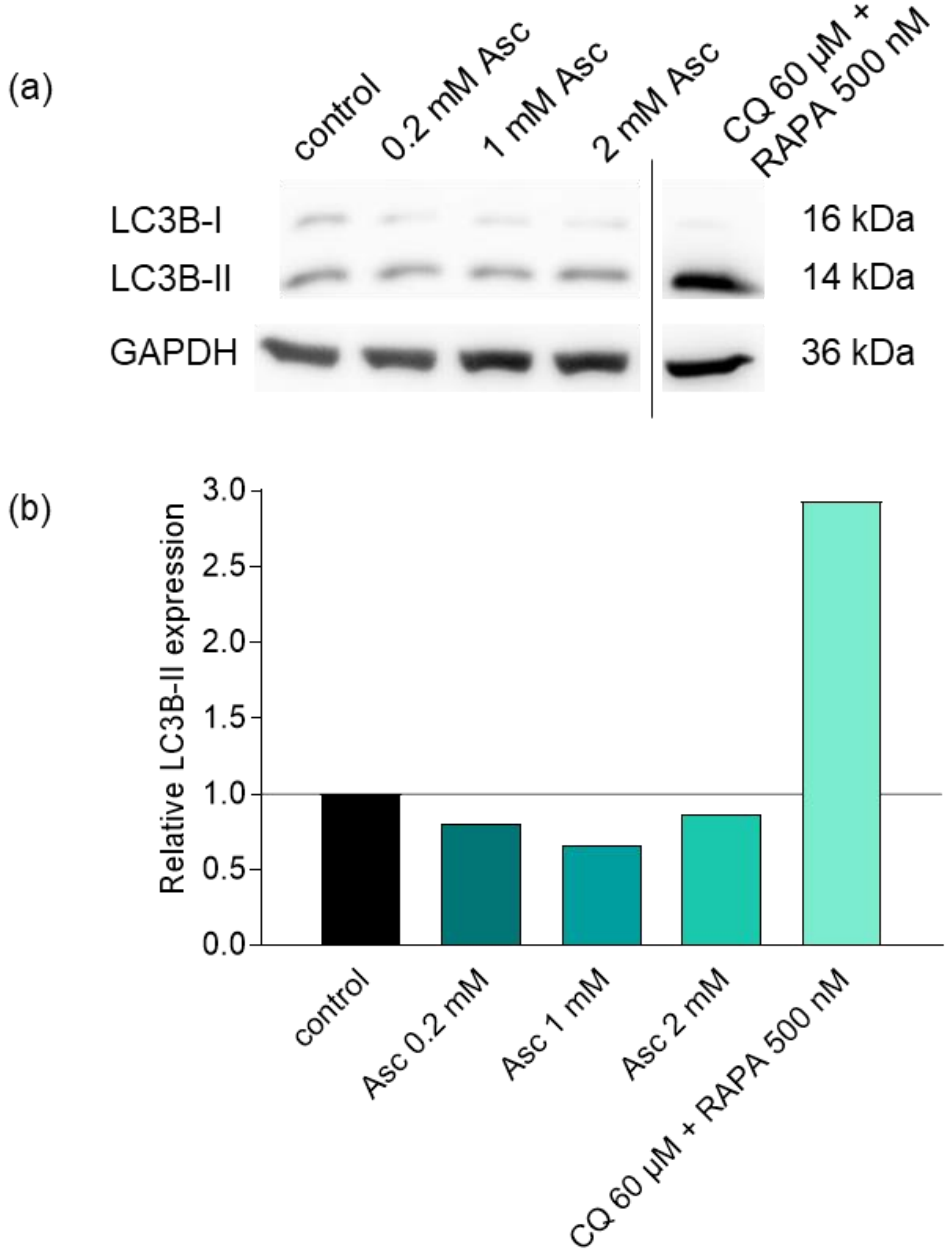

Figure S13: Western blot analysis of LC3B-II in cell lysates of SF268 cells treated with the indicated Asc concentrations (0.2 mM, 1 mM, and 2 mM) or CQ (60 µM) and RAPA (500 nM) as a positive control for 8 h. Equal protein loading was confirmed by GAPDH detection (**a**). Protein expression was quantified by densitometry (ImageJ version 1.54, National Institutes of Health, Maryland, USA), normalized to GAPDH and shown relative to the untreated control (**b**). Asc, ascorbate; CQ, chloroquine; GAPDH, glyceraldehyde-3-phosphate dehydrogenase; LC3B, microtubule-associated proteins 1A/1B light chain 3B; RAPA, rapamycin.
